# Hybridisation capture allows DNA damage analysis of ancient marine eukaryotes

**DOI:** 10.1038/s41598-021-82578-6

**Published:** 2021-02-05

**Authors:** L. Armbrecht, G. Hallegraeff, C. J. S. Bolch, C. Woodward, A. Cooper

**Affiliations:** 1 Australian Centre for Ancient DNA, School of Biological Sciences, Faculty of Sciences, The University of Adelaide, Adelaide, SA Australia; 2 Institute for Marine and Antarctic Studies, University of Tasmania, Hobart, TAS Australia; 3 Institute for Marine and Antarctic Studies, University of Tasmania, Launceston, TAS Australia; 4 Australian Nuclear Science and Technology Organisation, Lucas Heights, NSW Australia; 5 South Australian Museum, Adelaide, SA Australia

**Keywords:** Bioinformatics, Palaeoecology, Microbial biooceanography, Evolutionary ecology, Metagenomics, Eukaryote

## Abstract

Marine sedimentary ancient DNA (*sed*aDNA) is increasingly used to study past ocean ecosystems, however, studies have been severely limited by the very low amounts of DNA preserved in the subseafloor, and the lack of bioinformatic tools to authenticate *sed*aDNA in metagenomic data. We applied a hybridisation capture ‘baits’ technique to target marine eukaryote *sed*aDNA (specifically, phyto- and zooplankton, ‘Planktonbaits1’; and harmful algal bloom taxa, ‘HABbaits1’), which resulted in up to 4- and 9-fold increases, respectively, in the relative abundance of eukaryotes compared to shotgun sequencing. We further used the bioinformatic tool ‘HOPS’ to authenticate the *sed*aDNA component, establishing a new proxy to assess *sed*aDNA authenticity, “% eukaryote *sed*aDNA damage”, that is positively correlated with subseafloor depth. We used this proxy to report the first-ever DNA damage profiles from a marine phytoplankton species, the ubiquitous coccolithophore *Emiliania huxleyi*. Our approach opens new avenues for the detailed investigation of long-term change and evolution of marine eukaryotes over geological timescales.

## Introduction

Over the past decade marine sedimentary ancient DNA (*sed*aDNA) has become increasingly used to study past ocean ecosystems and oceanographic conditions. The novelty of using *sed*aDNA lies in its enormous potential to detect genetic signals of taxa that do and don’t fossilise—meaning that, in theory, it is possible to go beyond standard environmental proxies and facilitate reconstruction of past marine ecosystems across the entire food web. For example, *sed*aDNA has revealed relationships between past marine community composition and paleo-tsunami episodes in Japan over the past 2000 years^1^, oxygen minimum zone expansions in the temperate Arabian Sea region over 43 thousand years (kyr)^2^, and Arctic sea-ice conditions spanning 100kyr^3^. While the logistical challenge of acquiring undisturbed sediment cores from the deep seafloor remains, the field of *sed*aDNA research is rapidly advancing due to new ship-board core sampling procedures that allow far greater contamination control, and improvements in sample processing, sequencing technologies and bioinformatic tools^4^.

Among the huge diversity of marine eukaryotes, phytoplankton are particularly useful targets to study past ocean conditions. Phytoplankton are free-floating, unicellular microalgae fulfilling two important functions: (1) they form the base of the marine food web supporting virtually all higher trophic organisms (e.g., ^5^), and (2) are highly useful environmental indicators due to their sensitivity to changing physical and chemical oceanographic conditions^6^. After phytoplankton die, they sink to the seafloor where small proportions of their DNA are able to become entombed and preserved in sediments under favorable conditions, over time forming long-term records of past ocean and climate conditions. Using the small subunit ribosomal RNA gene (18S rRNA, a common taxonomic marker gene), we recently determined the fraction of marine eukaryote *sed*aDNA preserved in Tasmanian coastal sediments to be a mere 1.37% of the total *sed*aDNA pool^7^. A slightly higher proportion of eukaryote *sed*aDNA (and also higher diversity) may be captured by combining multiple taxonomic markers, e.g., the small and large subunit ribosomal RNA gene^8^. However, rather than analysing only part of the total *sed*aDNA pool (such as eukaryote marker genes within a large metagenomic dataset), a more cost-effective approach is to increase marine eukaryote *sed*aDNA yield through optimised extraction and sample processing that maximise sequencing of *sed*aDNA from the intended target organisms.

Metagenomic approaches extract and analyse the ‘total’ DNA in a sample (‘shotgun’ style), irrespective of the source organism, facilitating recovery of DNA sequences from any organism in proportion to their original presence in that sample. As a result, metagenomic approaches are well suited to the study of microbial and environmental ancient DNA (e.g., ^9–11^) including *sed*aDNA. This approach does not prescribe the target DNA fragment size and, importantly also preserves fragment size variability and damage patterns characteristic of ancient DNA that are vital to assess the authenticity of potential ancient genetic signals. However, achieving the sequencing depth required to detected and quantify organisms present at low relative abundance (such as eukaryotes in marine *sed*aDNA) can be very expensive. Therefore, there is a need for more targeted approaches that allow the detailed investigation of specific organisms, model or non-model, for which reads may otherwise go undetected in shotgun data.

**Figure 1:**
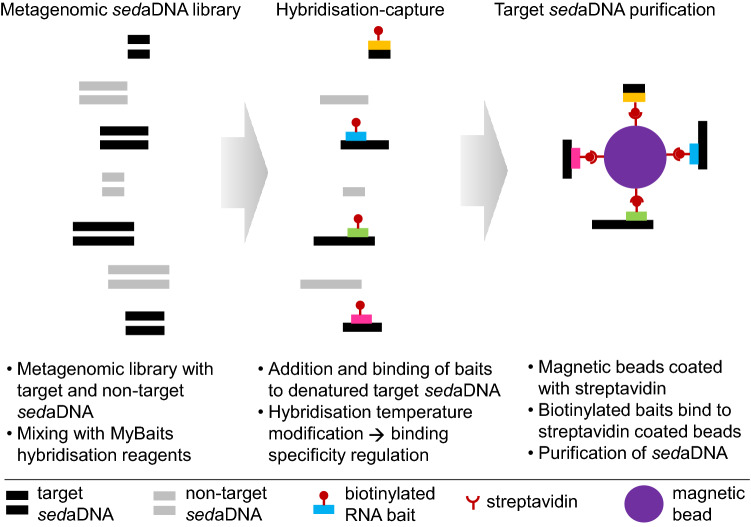
Schematic of hybridisation capture applied to marine sedimentary ancient DNA (*sed*aDNA). The three main steps are the preparation of a metagenomic *sed*aDNA library, hybridisation capture using RNA baits (in this study: Planktonbaits1 and HABbaits1) that are biotinylated, which enables binding of baits to streptavidin-coated magnetic beads (multiple baits per bead possible, schematic not to scale). For further technical details see Methods [and ^12,17^].

Hybridisation capture techniques are an increasingly popular method to focus the metagenomic analysis towards loci of interest, such as specific sequences to investigate particular groups of organisms^12,13^. Hybridisation capture uses short RNA probes (also called ‘baits’) designed to be complementary to DNA sequences of interest (e.g., taxonomic marker genes; Fig. 1). By binding to the target sequence, these genetic baits ‘capture’ DNA fragments from DNA extracts in a manner that preserves size variability, along with DNA damage patterns that can be used to examine whether sequences appear ancient. Additionally, careful bait design (i.e., selection of target sequences) and optimisation of the application protocol (e.g., hybridisation-temperature settings) allow differing levels of specificity in the capture process. While such ‘baits’ approaches have previously been used to investigate human, animal and even environmental DNA^14–16^, their application to marine sediments to capture *sed*aDNA from key primary producers and environmental indicator organisms (e.g., eukaryotic phytoplankton) remains untested.

The assessment of *sed*aDNA authenticity has been hindered by a lack of established approaches to identify and analyse DNA damage patterns of rare ancient microorganisms in metagenomic samples (such as eukaryotes in marine *sed*aDNA). For example, software commonly used to detect DNA damage patterns, such as ‘mapDamage’, computes nucleotide misincorporation and fragmentation patterns by mapping next-generation sequencing reads against a reference genome^18,19^. This requires high-quality modern reference genomes, or species where ancient DNA is available in sufficient quantity (e.g., animals^20^ or humans^21^), but neither is generally possible with marine eukaryote *sed*aDNA. There is a lack of high-quality reference sequences for the thousands of marine organisms occurring in the global ocean, and the threshold of ~ 250 reads per species required to analyse and plot DNA damage patterns in mapDamage^22^ is often not reached in *sed*aDNA. Recently, Hübler et al.^23^ developed a new bioinformatic tool HOPS—‘Heuristic Operations for Pathogen Screening’—based on the mapDamage algorithm, to identify and authenticate bacterial pathogens in ancient metagenomic samples and extract this information for further downstream analysis. In combination with hybridisation capture to generate a larger number of ancient eukaryote sequences, HOPS has the potential to allow the assessment of *sed*aDNA authenticity based on DNA damage profiles from key marine eukaryotes, even if only very few sequences are available (≥ 50 reads per species^23^).

In this work, we develop and apply two hybridisation capture bait sets for the first such analysis of marine sediments, targeting (i) marine phyto- and zooplankton very broadly for general paleoplankton assessment (Planktonbaits1), and selected key phytoplankton groups (especially, diatoms, dinoflagellates and coccolithophores) that are either highly abundant or the cause of harmful algal blooms (HABs) in our study region off the East Australian coast (HABbaits1). Based on samples from two coastal sediment cores collected near Maria Island, Tasmania, we demonstrate: (1) the effectiveness of Planktonbaits1 and HABbaits1 to maximise *sed*aDNA originating from eukaryote targets relative to shotgun data; (2) the authenticity of both shotgun- and baits-derived sequencing data via HOPS; (3) examine relationships between the ‘ancient’ DNA fraction and subseafloor depth through the development of a new *sed*aDNA proxy (‘% eukaryote *sed*aDNA damage’); and (4) generate the first-ever DNA damage profile for a keystone marine phytoplankton, the coccolithophore *Emiliania huxleyi*.

## Methods

### Samples

**Figure 2:**
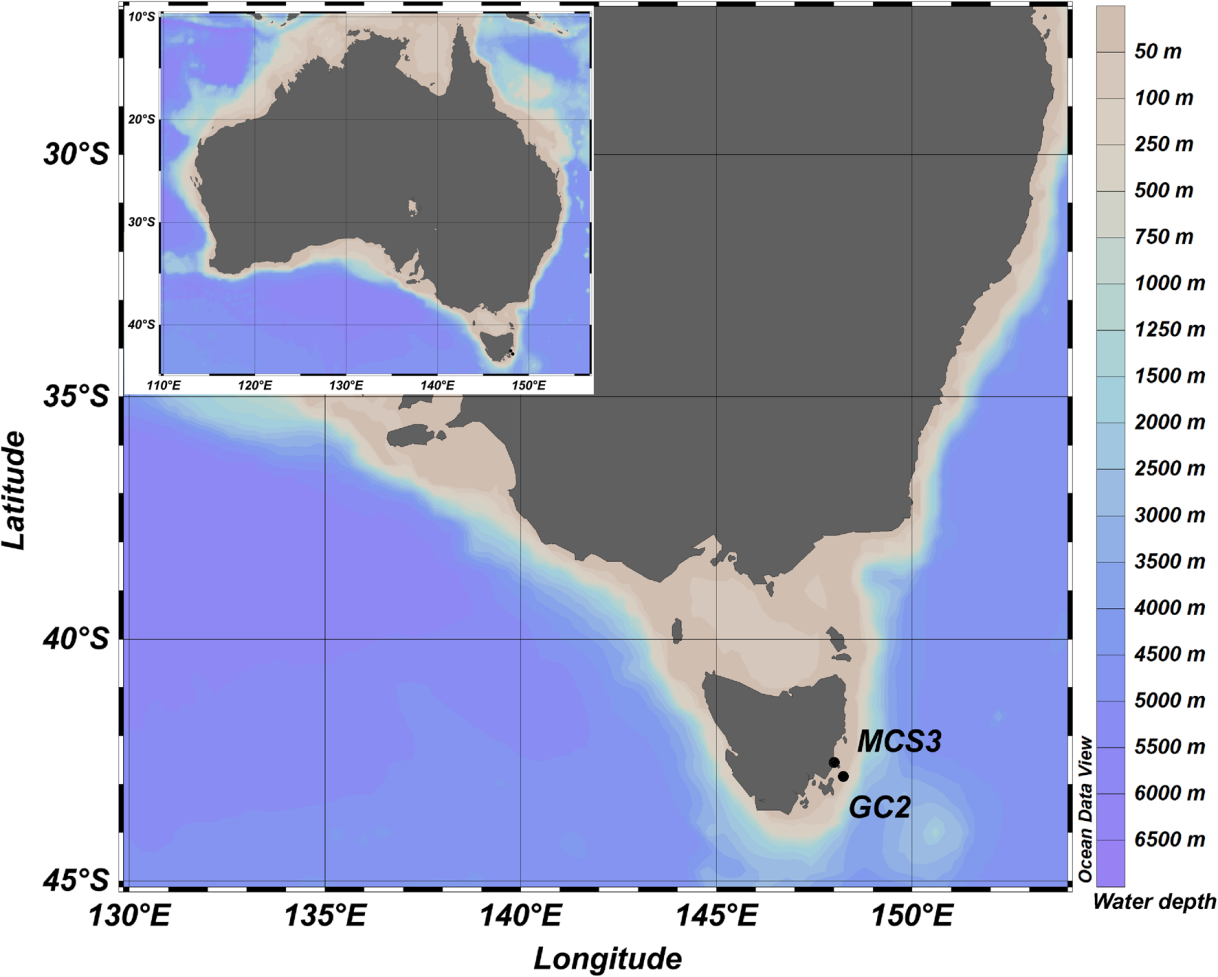
Map of coring sites, inshore (MCS3) and offshore (GC2) of Maria Island, Tasmania, South-East Australian Coast. Map created in ODV (Schlitzer, R., Ocean Data View, https://odv.awi.de, 2018).

Cores were collected during the *RV Investigator* voyage IN2018_T02 (19 and 20 May 2018, respectively, Fig. 2) to Tasmania, from sites in the Mercury Passage and Maria Island (Fig. 2). We collected one KC Denmark Multi-Core (MCS3, inner core diameter 10 cm, 36 cm long, estimated to cover the last ~ 145 years based on ^210^Pb dating at the Australian Nuclear Science and Technology Organisation (ANSTO, Lucas Heights, Sydney) in the Mercury Passage (MP, 42.550 S, 148.014 E; 68 m water depth), and one gravity core (GC2; inner core diameter 10 cm, 3 m long) offshore from Maria Island (42.845 S, 148.240 E; 104 m) composed of 2 sections; GC2A (bottom) and GC2B (top) estimated to cover the last ~ 8950 years based on ^210^Pb and ^14^C dating, ANSTO). The untreated cores were immediately sealed with plastic caps and sealed with duct-tape, stored initially on-board at 10 °C, followed by transport to and storage at 4 °C at ANSTO. To minimise contamination during core splitting and subsampling (October, 2018, ANSTO), we wiped working benches, sampling and cutting tools with bleach and 80% EtOH, changed gloves immediately when contaminated with sediment, and wore appropriate PPE at all times (gloves, facemask, hairnet, disposable lab gown). We removed the outer ~ 1 cm of the working core-half (working from bottom to the top of the core), then collected plunge samples by pressing sterile 15 mL centrifuge tubes (Falcon) ~ 2 cm deep into the sediment core centre at 5 cm depth intervals. All *sed*aDNA samples were immediately frozen at − 20 °C and transported to the Australian Centre for Ancient DNA (ACAD), Adelaide. For this study, a total of 30 samples were selected from both cores, representing ~ 2 cm depth intervals within the upper 36 cm of MCS3 and GC2, and ~ 20 cm depth intervals in GC2 downcore from 36 cm below seafloor (cmbsf).

### *Sed*aDNA extractions

We prepared *sed*aDNA extracts and sequencing libraries at ACAD's ultra-clean ancient (GC2) and forensic (MCS3) facilities following ancient DNA decontamination standards^24^. All sample tubes were wiped with bleach on the outside prior to entering the laboratory for subsampling. Our extraction method followed the optimised (“combined”) approach outlined in detail previously^7^, with a minor modification in that we stored the final purified DNA in TLE buffer (50 μL Tris HCL (1 M), 10 μL EDTA (0.5 M), 5 mL nuclease-free water) instead of customary Elution Buffer (Qiagen) (see Supplementary Material Methods). To monitor laboratory contamination, we used extraction blank controls (EBCs) by processing 1–2 (depending on the extraction-batch size) empty bead-tubes through the extraction protocol. A total of 30 extracts were generated from sediment samples and 7 extracts from EBCs.

### RNA-baits design

We designed two RNA hybridisation bait-sets, one targeting phyto- and zooplankton for a more detailed overview of plankton diversity (hereafter ‘Planktonbaits1’), and one targeting specific plankton organisms and their predators to enable detailed investigation of HABs, especially those caused by dinoflagellates, in coastal marine ecosystems (hereafter, ‘HABbaits1’). Planktonbaits1 was based on 18S-V9 and 16S-V4 sequences of major phyto- and zooplankton groups, whereas we designed HABbaits1 from a collection of LSU, SSU, D1-D2-LSU, COI, rbcL and ITS sequences for specific marine target organisms often associated with HABs in our study region (Table 1).

**Table 1:** Planktonbaits1 and HABbaits1.

Bait set	Target taxa	Targeted gene/gene region	Database from which sequences were acquired
Planktonbaits1	Ciliophora, MALV, Dinophyceae, Archaeplastida, Euglenida, Telonemia, Haptophyta, Cryptophyta, Katablepharidophyta, Chlorarachnea, Phaeodarea, Foraminifera, Acantharea, Other_Radiolaria, RAD, Collodaria, MAST, Bicoeca, MOCH, Raphidophyceae, Pinguiophyceae, Phaeophyceae, Chrysophyceae-Synurophyceae, Pelagophyceae, Dictyochophyceae, Bolidophyceae-and-relatives, Bacillariophyta	18S-V9	W2_PR2_V9^25^
	*Trichodesmium erythraeum, Prochlorococcus marinus, Synechococcus* sp.	16S-V4	SILVA^26^ https://www.arb-silva.de/
HABbaits1	**Dinoflagellates**		LSU: SILVA; SSU: PR2^27^ or NCBI (https://www.ncbi.nlm.nih.gov/); D1D2: PHYTOPK28S-D1D2^28^; ITS: BOLD^29^ (http://www.boldsystems.org/) or NCBI; rbcL: BOLD; COI: BOLD or NCBI
	*Alexandrium tamarense* Group 1 *(A. catenella)*	LSU, D1D2, ITS, COI	
	*Alexandrium tamarense* Group 2 *(A. mediterraneum)*	LSU, D1D2, ITS, COI	
	*Alexandrium tamarense* Group 3 *(A. tamarense)*	LSU, D1D2, ITS, COI	
	*Alexandrium tamarense* Group 4 *(A. pacificum)*	LSU, D1D2, ITS, COI	
	*Alexandrium tamarense* Group 5 *(A. australiense)*	LSU, D1D2, ITS, COI	
	*Gymnodinium catenatum*	LSU, D1D2, ITS, COI	
	*Noctiluca scintillans*	LSU, D1D2	
	*Tripos (Ceratium) furca*	LSU, SSU, D1D2	
	*Tripos (Ceratium) fusus*	LSU, SSU, D1D2, COI	
	*Tripos* sp. (genus)	SSU	
	*Tripos muelleri*	LSU, SSU	
	**Diatoms**		
	*Pseudo-nitzschia* sp. (genus)	LSU, D1D2, SSU, ITS	
	*Pseudo-nitzschia cuspidata*	LSU, D1D2, ITS, rbcL	
	*Pseudo-nitzschia pungens*	LSU, D1D2, ITS, rbcL	
	**Haptophytes**		
	*Emiliania huxleyi*	LSU, D1D2, rbcL, COI	
	**Cnidarians**		
	*Aurelia* spp.	LSU, D1D2, ITS, COI	
	*Cyanea* spp.	LSU, ITS, COI	
	*Physalia*	LSU, ITS, COI	
	**Molluscs**		
	*Crassostrea gigas*	LSU, D1D2, ITS, COI	
	*Ostrea angasi*	LSU, COI	
	*Mytilus galloprovincialis*	LSU, D1D2, ITS, COI	
	*Modiolus* spp.	LSU, D1D2, ITS, COI	
	**Genes involved in toxin production**		
	*SxtA*		

Target taxa of Plankton- and HABbaits1 genes/gene regions and source databases. For HABbaits1, all listed databases were searched for each gene (region) per target taxon, and, if available, the longest sequence was selected and included.

#### Planktonbaits1

To design Planktonbaits1 we downloaded the W2_V9_PR2 database^25^ (containing 18S-V9 rDNA and rRNA sequences of marine protists and their predators, downloaded on 30 July 2018), deduplicated using Geneious software (Geneious NZ), and filtered the remaining sequences to keep only those from major phyto- and zooplankton groups (Table 1). In collaboration with Arbor Biosciences, USA, we designed RNA baits based on these 15,035 target sequences by masking any repeating Ns (i.e.*,* any consecutive Ns that were < 10 in a row were converted to Ts, with ultimately 0.1% masked), padding short targets to 84 nucleotides (nt) (i.e.*,* any target less than 84 nt was padded with Ts up to 84 nt in length). This procedure provided 41,798 raw baits of 80 nt with 3 × tiling (creating an even coverage, i.e.*,* one bait every ~ 27 nt). The raw baits were BLASTed against ArborBioscience’s in-house RefSeq database containing 5584 bacterial genome and plasmid sequences (downloaded from NCBI, May, 2018), and any baits leading to hits were removed (except for 785 loci from cyanobacterial taxa that we intended to keep, see below). This filtering step provided 36,836 baits, which were collapsed into 15,942 final baits (i.e.*,* eliminating redundancy based on identity and overlap; using > 83% overlap, and > 95% identity). We added five 16S-V4 rRNA sequences (the prokaryotic equivalent of the small subunit ribosomal rRNA gene) of common marine cyanobacteria (one *Trichodesmium erythraeum* sequence, and two *Prochlorococcus marinus* and *Synechococcus* sp. sequences each), acquired from the SILVA database^26^; Table 1). To check and ensure target-taxon specificity, these five cyanobacterial sequences were mapped against a non-target sequence (*Escherichia coli* 16S RefSeq sequence NR_114042.1), then reverse-transcribed to DNA, and BLASTed to the same NCBI RefSeq database described above. BLAST hits of < 60 bp alignment length and < 80% identity were removed, and only those baits with < 50 BLAST hits were kept, resulting in 10 cyanobacterial baits. Consequently, Planktonbaits1 contained a total of 15,952 RNA baits targeting the 18S-V9 region of a broad diversity of phytoplankton and their predators and the 16SV4 region of three cyanobacteria.

#### HABbaits1

To design HABbaits1 we manually collated a total of 805 LSU, SSU, D1-D2-LSU, COI, rbcL and ITS sequences for specific marine target organisms often associated with harmful algal bloom events in our study region, primarily dinoflagellates but also certain diatoms, a coccolithophore, jelly- and shellfish and the saxitoxin A4 gene, involved in paralytic shellfish toxin production by the dinoflagellates *Gymnodinium catenatum* and some species of the genus *Alexandrium* (Table 1)*. *As with Planktonbaits1, we worked in collaboration with Arbor Biosciences, USA, to design RNA baits based on the collated sequences (converting consecutive (< 10) Ns to Ts and RNA sequences to DNA, masking input sequences for simple repeats (0.4%)), attaining 23,064 raw 80 nt baits (using 3 × tiling, as for Planktonbaits1, see section “Planktonbaits1”). Each bait candidate was BLASTed against three target genomes (the oyster *Crassostrea gigas,* coccolithophore *Emiliania huxleyi,* mussel *Mytilus galloprovincialis*), and four non-target genomes (diatoms *Fragilariopsis cylindrus, Phaeodactylum tricornutum,* dinoflagellate *Symbiodinium minutum,* diatom *Thalassiosira pseudonana,* jellyfish *Clytia hemisphaerica*), and a hybridisation melting temperature (T_m_)* was estimated for each hit assuming standard myBaits buffers and conditions (T_m_ is defined as the temperature at which 50% of molecules are hybridised). For each target bait candidate, one BLAST hit with the highest T_m_ was first discarded from the results (allowing for 1 hit in the genome), and only the top 500 hits (by bit score) were considered. Based on the distribution of remaining calculated T_m_'s, we filtered out non-specific baits using stringent (only specific baits pass) criteria (i.e., bait candidates pass if they satisfy one of these conditions: (a) no hits with T_m_ above 60 °C, (b) ≤ 2 hits 62.5–65 °C, (c) ≤ 10 hits 62.5–65 °C and at least 1 failing flanking bait, (d) ≤ 10 hits 62.5–65 °C, 2 hits 65–67.5 °C, and < 2 passing flanking baits, (e) ≤ 2 hits 62.5–65 °C, 1 hit 65–67.5 °C, 1 hit 70 °C or above, and < 2 passing flanking baits. Bait candidates were removed when a hit was determined after BLASTing them against the non-target genomes. This highly stringent filtering procedure for HABbaits1 was applied to ensure maximum target-specificity of our selected HAB species, and resulted in a total of 15,310 baits for this set.

### Library preparations and hybridisation capture

We prepared sequencing libraries from all DNA extracts following previously established protocols^11^. Briefly, a 20 µL aliquot of DNA was repaired (15 min, 25 °C) in a 40 µL reaction using T4 DNA polymerase (New England Biolabs). After purifying the DNA (MinElute Reaction Cleanup Kit, Qiagen), a ligation step followed (T4 DNA ligase, Fermentas) in which truncated Illumina-adapter sequences containing two unique 5 base-pair (bp) barcodes were attached to the double-stranded DNA^30^ (60 min, 22 °C). DNA purification (MinElute Reaction Cleanup Kit, Qiagen) was performed, followed by a fill-in reaction with adapter sequences (Bst DNA polymerase, New England Biolabs; 30 min, 37 °C, with polymerase deactivation for 10 min, 80 °C). After barcode ligation, we prepared metagenomic shotgun libraries following a previously described protocol^7^, with slight modifications described in Supplementary Material Methods.

For sequencing library preparations for the hybridisation capture we followed the MyBaits Manual^17^ (Arbor Biosciences, USA). The latter recommends a minimum of 100 ng DNA in 7 µL as input for hybridisation capture reactions, however, based on pilot trials with three marine sediment samples (not shown), we determined that this minimum input can be reduced to ~ 50 ng if *sed*aDNA concentrations are very low, as was the case for our samples. To achieve at least ~ 50 ng input DNA in 7 µL, we re-amplified remaining *sed*aDNA of most of our shotgun libraries (cleaned post-IS7/IS8 PCR products) in a second IS7/IS8 PCR (one 75 µL reaction with 9 µL DNA input per sample, using 10 amplification cycles and the same reagent composition as for shotgun IS7/IS8 PCRs, see Supplementary Material Methods). We combined the barcoded EBCs (1 µL each, using a 1 in 10 dilution of EBC_A24029 due to its comparably high DNA concentration relative to the other EBCs) in one PCR reaction (7 µL EBC DNA template total) for the downstream enrichments. After re-amplification, the *sed*aDNA was cleaned using AxyPrep magnetic beads (1:1.8 library:beads) and quantified using Qubit DNA assays. Samples for which the initial IS7/IS8 PCR provided comparatively high DNA concentrations were not re-amplified prior to hybridisation capture. Using this procedure, we generated 62.53 ± 25.92 ng of DNA (23.24–171.75 ng; 0.07 ng for the EBC pool) for use as input material for the hybridisation capture with Planktonbaits1 and HABbaits1.

Hybridisation capture followed the MyBaits Manual^17^ with slight modifications. In the Hybridisation Mix (“HYBs”) we used 3 µL baits per reaction, and in the Blockers Mix (“LIBs”) we used the blockers Nimblegen SeqCapEZ (a plant repetitive elements blocker), Block O and Block A (Salmon Sperm DNA and P5/P7 block, respectively, both provided with the MyBaits kit), and we added 7 µL of DNA template. We combined LIBs and HYBs per sample in a Thermocycler (Thermoscientific) once the latter had been at hybridisation temperature for 5 min. For Planktonbaits1 we set the hybridisation temperature to 60 °C as per the manufacturer’s recommendation for short and damaged DNA molecules, and the hybridisation reaction to 40 h. For HABbaits1, we set the hybridisation temperature to 65 °C for the first 3 h to favour highly specific binding, followed by a decrease to 60 °C for the remaining 37 h of the hybridisation capture reaction. We prepared the beads for batches of 8 reactions in 1.7 mL tubes by washing the beads twice with binding buffer, then adding binding buffer and 48 µL yeast tRNA (= 480 µg per 240 mL beads) in a third washing step, followed by brief vortexing and incubation of the solution on a rotary mixer (30 min, room temperature), pelleting on a magnetic rack, and two more washes with binding buffer. We performed bead-hybrid binding for 20 min at 60 °C, with agitation by pipette-mixing, and briefly centrifuging to collect after 5 min. Subsequent washes and library resuspensions (in 40 µL Buffer EBT (EB (Qiagen) with 0.05% Tween20 (Sigma Aldrich)) were performed as per protocol for non-KAPA HiFi HotStart polymerase amplification (incubation at 95 °C, pelleting of beads and collection of *sed*aDNA containing buffer EBT).

GaII Indexing PCRs (using different indices for HABbaits1 and Planktonbaits1) were performed as for shotgun sequencing libraries (Supplementary Material Methods), but we used one 100 µL reaction and 16 amplification cycles per sample. Initially, we used 12 and 24 µL hybridisation capture *sed*aDNA template generated from MCS3 and GC2S1 samples as we assumed relatively high and low DNA concentrations, respectively. For samples GC2B 15–16.5 cm, GC2B 75–76.5 cm and GC2A 65–66.5 cm we used 12 µL DNA template due to previous experimental trials. Following amplification, very low DNA concentrations were determined for all HABbaits1 samples and Planktonbaits1 samples MCS3 2–3.5 cm, MCS3 4–5.5 cm, GC2B 85–86.5 cm and GC2A 75–76.5 cm. Therefore, we used the remaining hybridisation capture material (26 µL from MCS3 and 14 µL from GC2S1 samples) from these samples in a second GaII Indexing PCR (100 ul reaction, 16 cycles). We combined the initial and supplementary GaII PCR products per sample and concentrated to 15 µL (20 min, 45 °C) using a CentriVap concentrator (Labconco, USA). To clean the PCR products we used AxyPrep beads (1:1.1 PCR products:beads), eluted the beads in 30 µL nuclease-free H_2_O and assessed DNA quantity and quality through TapeStation. We prepared an equimolar (6 nM) sequencing pool from all samples, which we concentrated using CentiVap (45 min, 45 °C) to 110 µL, and cleaned using AxyPrep beads (1:1.1 sequencing pool:beads). Following DNA quantity and quality assessment using Qubit, TapeStation, and Fragment Analyzer, we performed one more AxyPrep clean-up (same ratio). We ran final DNA quantity and quality checks via Fragment Analyser and qPCR, and prepared a sequencing pool (mean fragment size 225 bp, 2.75 nM) for submission to Illumina sequencing (HiSeq XTen, 2 × 150 bp cycle). Sequencing was performed at the Australian Cancer Research Foundation Cancer Genomics Facility & Centre for Cancer Biology, Adelaide, Australia, and at the Garvan Institute of Medical Research, KCCG Sequencing Laboratory (Kinghorn Centre for Clinical Genomics), Darlinghurst, Australia.

### Data analysis

#### Bioinformatics

Bioinformatic processing and filtering of the sequencing data, hereafter referred to as datasets ‘Shotgun’, ‘Planktonbaits1’ and ‘HABbaits1’, followed established protocols previously described^7^, with the exception that we used the NCBI Nucleotide database (ftp://ftp.ncbi.nlm.nih.gov/blast/db/FASTA/nt.gz, downloaded November 2019) as the reference database to align our *sed*aDNA sequences to (allowing us to run all three datasets against the same database; see Supplementary Material Methods). All species detected in EBCs (Supplementary Material Table 1) were subtracted from the sample data, and hereafter the term ‘samples’ refers to sediment-derived data post-EBC subtraction. For each dataset (Shotgun, Planktonbaits1 and HABbaits1), we used MEGAN6 Community Edition v6.18.10 to rank our assigned reads by domain and exported these read counts. We determined relative abundances per domain per sample, and the average and standard deviation per domain across all samples from MCS3 and GC2S1 (separately for each site due to relatively high variability in relative abundance between them, see results). To quantify the increase in the proportion of our target domain Eukaryota using Planktonbaits1 and HABbaits1 relative to Shotgun, we determined the ratio between the average relative abundance per domain between Planktonbaits1:Shotgun, and HABbaits1:Shotgun.

#### Ancient DNA authenticity assessment and damage analysis

To assess the authenticity of our Shotgun, Planktonbaits1 and HABbaits1 *sed*aDNA we ran the ‘MALTExtract’ and ‘Postprocessing’ tools of the HOPS v0.33-2 pipeline^23^. The latter included the use of the NCBI mapping and NCBI tree files (13 Nov 2019) provided with HOPS (https://github.com/rhuebler/HOPS/tree/external/Resources). Configurations deviating from the default HOPS settings included topMaltEx = 0.10, minPIdent = 95, meganSummary = 1, and destackingOff = 1. We processed each dataset using the ‘def_anc’ mode, which provided results for all filtered reads (‘default’) as well as all reads that had at least one damage lesion in their first 5 bases from either the 5′ or 3′ end (‘ancient’)^23^. Generally, HOPS determines DNA damage patterns separately for individual taxa, i.e., requires an input list of target taxa for which to compare the *sed*aDNA sequences identified in our samples to their modern references. We used two taxa screening lists with the aim to generate *sed*aDNA damage profiles for a representative regional eukaryotic plankton species: (a) the first taxa list simply specified the single word ‘Eukaryota’, which prompts HOPS to run through each eukaryote taxon identified, thereby allowing a general assessment of the amount of eukaryote sequences categorised as ‘default’ or ‘ancient’ in each of our samples and EBCs; and (b) our second taxa list contained the names of the specifically selected marine organisms included in HABbaits1, which are known to be common in our Tasmanian study region (Table 1).

Subsequently to running (a) we used the HOPS-generated ‘RunSummary’ output (containing read counts per taxon classified as either ancient or default) to determine eukaryote-derived percent damage in each dataset (Shotgun, Planktonbaits1 and HABbaits1). Separately for each dataset, we subtracted all taxa with no read counts in both the ancient or default output, and taxa for which read counts were determined in either the ancient or default output (or both) of EBCs (Supplementary Material Table 2). Next, we summed the number of eukaryote reads per sample for ancient and default outputs (total reads) and calculated the proportion between these ancient and default totals, with the proportion of ancient reads providing a ‘% eukaryote *sed*aDNA damage’ measure per sample. Subsequent to running (b) on all three datasets (Shotgun, Planktonbaits1 and HABbaits1)*,* we used the MaltExtract Interactive Plotting Application (MEx-IPA, by J. Fellows Yates; https://github.com/jfy133/MEx-IPA) to visualise *sed*aDNA damage profiles (ancient reads only) of the target phytoplankton taxa (Table 1), however, sufficient ancient reads to generate these profiles for all samples in all three datasets were only consistently achieved for the coccolithophore *Emiliania huxleyi*.

#### Statistics

To determine relationships between % eukaryote *sed*aDNA damage and subseafloor depth and test the ‘% eukaryote *sed*aDNA damage’ measure’s validity as *sed*aDNA authenticity proxy, we performed two-tailed Pearson correlation analyses between the % eukaryote *sed*aDNA damage determined in Shotgun, Planktonbaits1 and HABbaits1 (n = 27 each, excluding 3 samples, see section “Proportions of Eukaryota in Shotgun, Planktonbaits1 and HABbaits1”) and subseafloor depth using the software PAST^31^.

## Results

### Proportions of Eukaryota in Shotgun, Planktonbaits1 and HABbaits1

After filtering, we retained between 4.6 (GC2A 15–16.5 cm) and 16.2 M (GC2B 5–6.5 cm) reads per sample for Shotgun, between 0.1 (MCS3 4–5.5 cm) and 4.6 M (GC2A 115–116.5 cm) reads per sample for Planktonbaits1 and between 0.2 (GC2A 45–46.5 cm) and 2.8 M (GC2A 115–116.5 cm) reads for HABbaits1. We retrieved no data (or nearly no data, thus not representative) for 3 out of 30 samples and these samples were excluded from downstream processing. The 3 samples were MCS3 0–1.5 cm with Shotgun, GC2B 115–116.5 cm with Planktonbaits1 and HABbaits1, and GC2A 85–86.5 cm with HABbaits1—likely due to low template DNA concentrations (see Supplementary Material). Our EBCs for Shotgun, Planktonbaits1 and HABbaits1 detected a total of 121, 69 and 28 eukaryote taxa (Supplementary Material Table 1). Of note was the identification of *Cyprinus carpio* (European/Common carp) sequences in all Shotgun, most Planktonbaits1 and three HABbaits1 EBCs. Being a freshwater species unlikely to occur offshore Tasmania, we considered cross-contamination from samples to EBCs irrespective of extraction batch unlikely. Most likely, this is a reagent-derived contaminant as we have detected this species in extraction blanks of unrelated datasets (not shown). Here, *C. carpio* was removed from downstream analyses and does not impact on our results, however, this finding emphasises the importance of sequencing controls and filtering *sed*aDNA data accordingly to remove contaminants.

**Figure 3:**
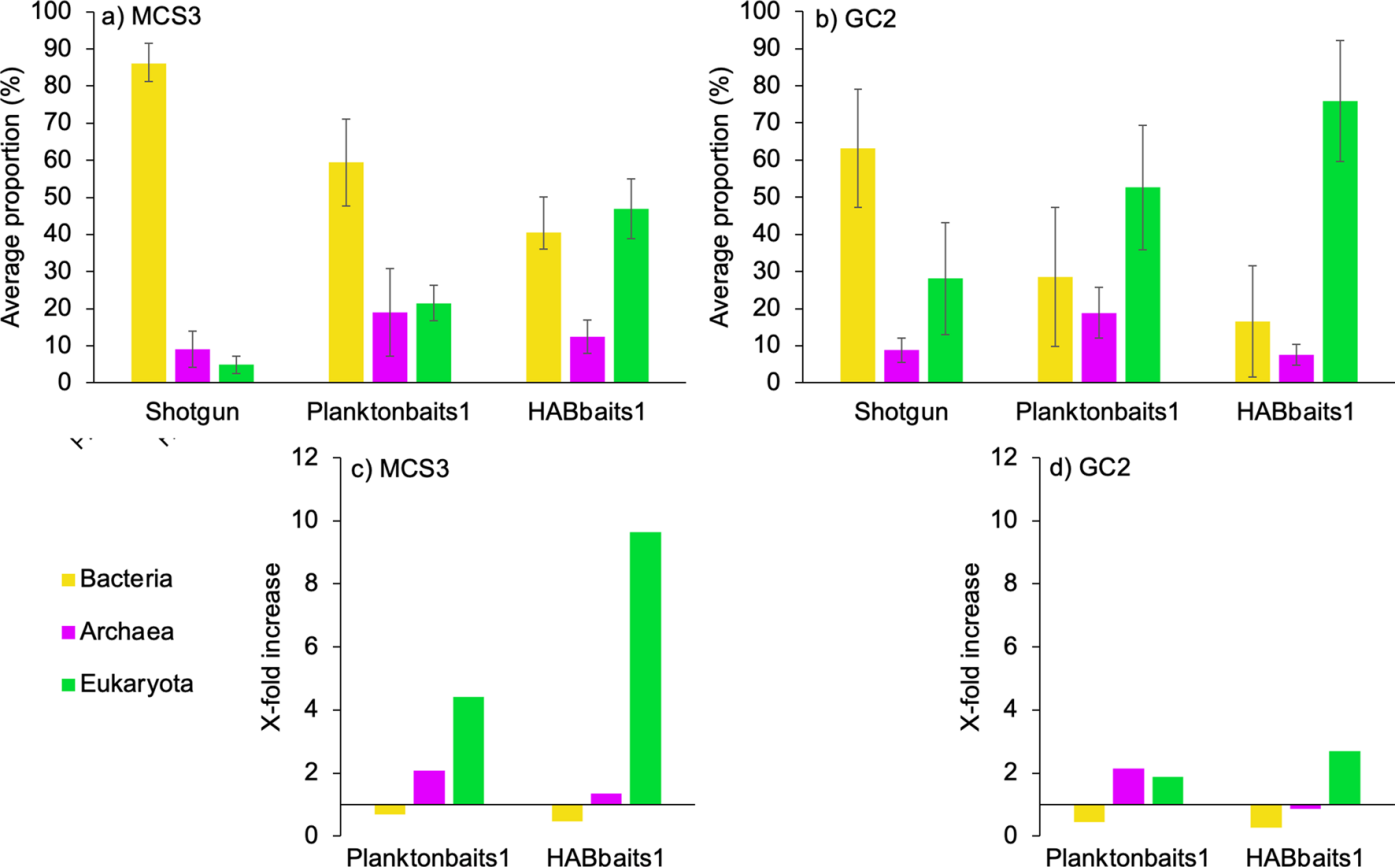
Proportions of reads assigned to Bacteria, Archaea and Eukaryota using Shotgun, Planktonbaits1 and HABbaits1. (**a**,**b**) Average proportion of reads and standard deviation across inshore MCS3 (n = 9) and offshore GC2 (n = 18) samples, respectively. (**c**,**d**) Increase in the proportion of Bacteria, Archaea and Eukaryota in Planktonbaits1 and HABbaits1 relative to Shotgun for MCS3 and GC2 samples, respectively, based on average proportions shown in (**a**,**b**).

Based on alignments using the NCBI Nucleotide database, the majority of Shotgun reads were assigned to Bacteria (86 ± 5% and 63 ± 16% for MCS3 and GC2, respectively; Fig. 3a,b), and a relatively small portion to Eukaryota (5 ± 2% and 28 ± 15% for MCS3 and GC2, respectively, Fig. 3a,b). This small proportion of Eukaryota increased to 21 and 53% in MCS3 and GC2 using Planktonbaits1 (4.4 × and 1.9 × over Shotgun, respectively), and 47 and 76% in MCS3 and GC2 using HABbaits1 (9.6 × and 2.7 × over Shotgun respectively) (Fig. 3). Planktonbaits1 and HABbaits1 were efficient in the targeted enrichment of Eukaryota *sed*aDNA from marine sediments, with comparatively little ‘bycatch’ of Bacteria and Archaea (i.e., a decrease in the proportion of Bacteria and a < 2.1 × increase in Archaea relative to Shotgun; Fig. 3c,d). Planktonbaits1 included three cyanobacterial targets, therefore, some capture of bacterial sequences was expected; less than Shotgun but more than HABbaits1 (Fig. 3a,b).

### Assessment of sedaDNA authenticity

**Figure 4:**
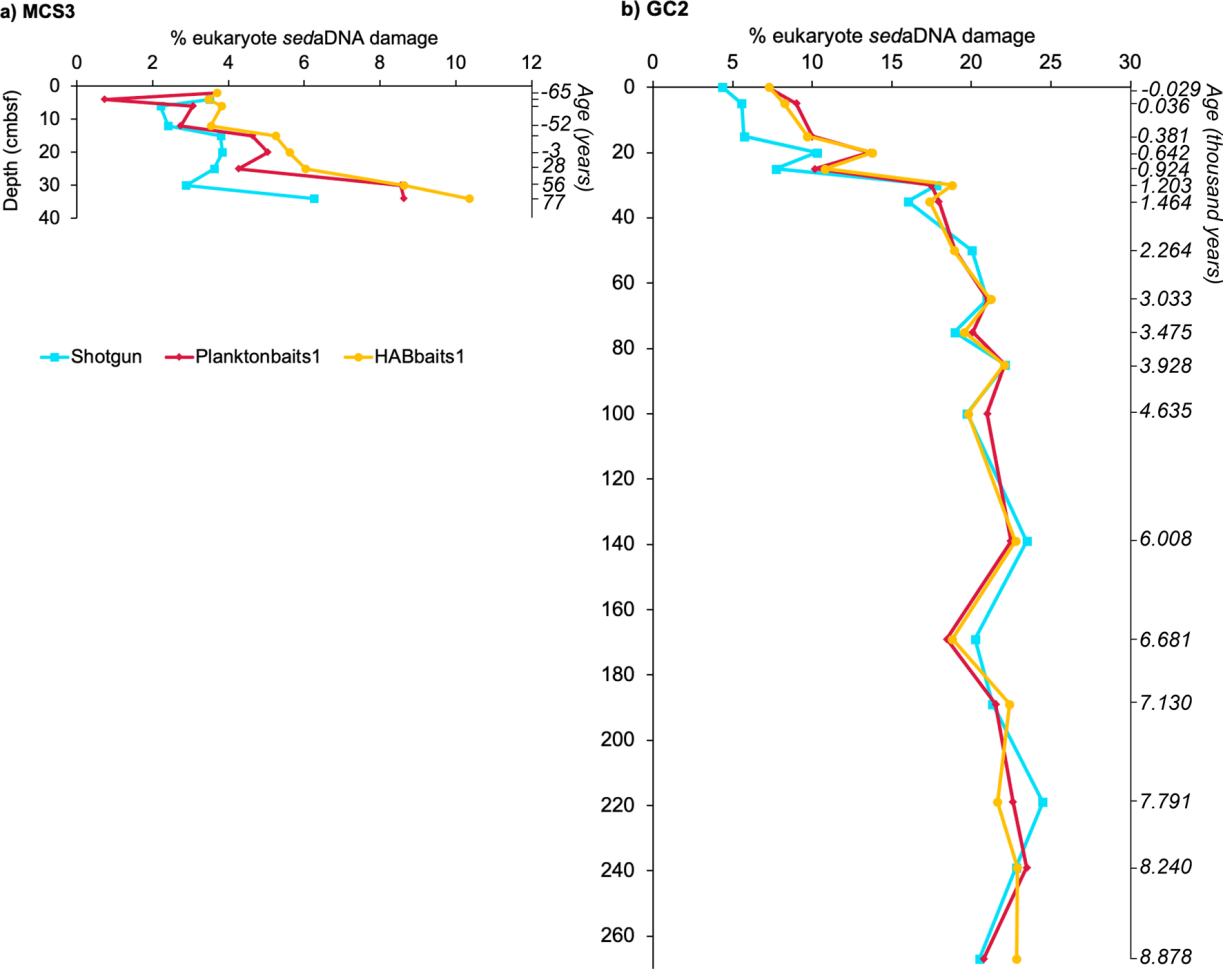
Eukaryote *sed*aDNA damage with subseafloor depth. Shown is the increase in % eukaryote *sed*aDNA damage with depth (centimetres below seafloor, cmbsf) in both (**a**) MCS3, (**b**) GC2. See Table 2 for correlation between % eukaryote *sed*aDNA damage per dataset (Shotgun, Planktonbaits1, HABbaits1) and depth, and amongst the datasets. Age of sediment cores is recorded relative to the year 1950 (reference date for radiocarbon dating), i.e.*,* MCS3 is ~ 145 years old (**a**) and GC2 ~ 8946 years old (**b**), and negative ages indicate years post-1950 (e.g.*,* an age of -65 equals the year 2015).

For both inshore MCS3 and offshore GC2, the ‘% eukaryote *sed*aDNA damage’ determined per sample increased with sub-seafloor depth for each of the three datasets Shotgun, Planktonbaits1 and HABbaits1 (Fig. 4). At the seafloor surface, we determined *sed*aDNA damage of ≤ 4% at MCS3, and between 4 and 10% at GC2, which, at both sites, slightly increased with depth until ~ 25 cmbsf (to about ~ 10% and 10–15% at MCS3 and GC2, respectively), before a steeper increase between ~ 25 and 35 cmbsf, and, in offshore GC2, remained relatively stable at ~ 20 and 25% below 35 cmbsf (> 1400 years of age). Correlation analyses showed that this increase of the % eukaryote *sed*aDNA damage with increasing subseafloor depth was highly significant for each dataset (Table 2). Additionally, the % eukaryote *sed*aDNA damage of the three different datasets (Shotgun, Planktonbaits1 and HABbaits1) were significantly positively correlated with each other, indicating that the original proportions of eukaryote *sed*aDNA damage patterns preserved in Shotgun were maintained in our hybridisation capture approach using both Planktonbaits1 and HABbaits1.

**Table 2:** Summary statistics of correlation analysis between % eukaryote *sed*aDNA damage and subseafloor depth.

	Depth (cmbsf)	Shotgun % damage	Planktonbaits1% damage	HABbaits1% damage
MCS3				
Depth (cmbsf)		0.01361	0.00241	0.00026
Shotgun % damage	*0.77773*		0.00032	0.00229
Planktonbaits1% damage	*0.86805*	*0.92695*		0.00018
HABbaits1 A:D	*0.93138*	*0.87002*	*0.93812*	
GC2				
Depth (cmbsf)		0.00064	0.00072	0.00040
Shotgun % damage	*0.72638*		9.84E-14	7.35E-13
Planktonbaits1% damage	*0.72182*	*0.98541*		1.89E-14
HABbaits1% damage	*0.74356*	*0.98121*	*0.98815*	

Pearson correlation coefficients *r* (in italics, lower matrix triangle) and corresponding two-tailed probability that *r* is uncorrelated (upper triangle of matrix; i.e.*,* all values < 0.05 denote a significant correlation) between subseafloor depth (cmbsf) and Shotgun, Planktonbaits1 and HABbaits1% eukaryote *sed*aDNA damage (n = 27 each).

### DNA damage profiles of the marine coccolithophore *Emiliania huxleyi*

**Figure 5:**
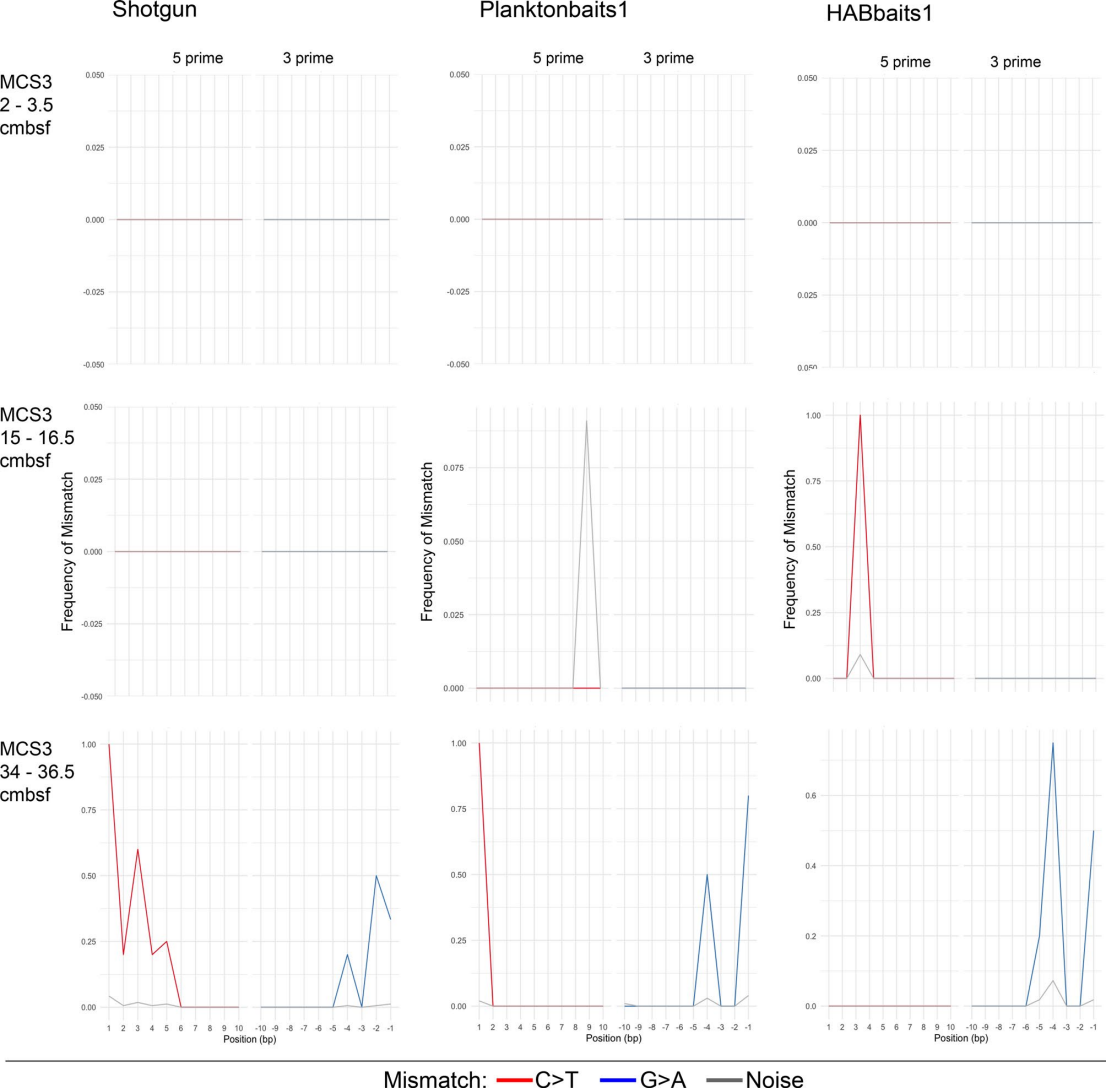
*Sed*aDNA damage profiles of *Emiliania huxleyi* in MCS3*. E. huxleyi sed*aDNA damage profiles (frequency of mismatch against base pair position) per sample for Shotgun, Planktonbaits1 and HABbaits1 in MCS3. The red and blue lines denote C > T substitutions in 5′ direction and G > A substitutions in 3′ direction, respectively, for all ancient alignments. Grey lines denote estimated noise^23^. Shown are selected samples only (top, mid-depth and bottom sample), for damage profiles of all samples see Supplementary Material Fig. 1.

**Figure 6:**
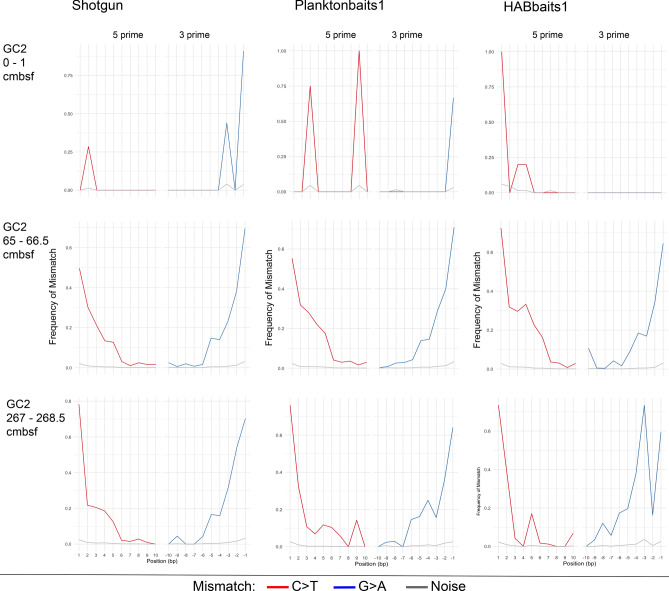
*Sed*aDNA damage profiles of *Emiliania huxleyi* in GC2*. E. huxleyi sed*aDNA damage profiles (frequency of mismatch against base pair position) per sample for Shotgun, Planktonbaits1 and HABbaits1 in GC2. The red and blue lines denote C > T substitutions in 5′ direction and G > A substitutions in 3′ direction, respectively, for all ancient alignments. Grey lines denote estimated noise^23^. Shown are selected samples only (top, mid-depth and bottom sample), for damage profiles of all samples see Supplementary Material Fig. 2.

The *sed*aDNA damage analysis provided DNA damage profiles for most of the target taxa on our selected taxa list (taxa list ‘*b*’). However, the number of ancient sequences assigned to the ubiquitous coccolithophore *Emiliania huxleyi* was much higher, allowing the generation of more detailed *sed*aDNA damage profiles, consistently across all three datasets. Ancient *E. huxleyi* sequences ranged from a total of 0–10 reads in inshore MCS3 and 5–2651 in offshore GC2 for Shotgun, from 0 to 7 in MCS3 and 1 to 947 in GC2 for Planktonbaits1, and from 0 to 11 in MCS3 and 1 to 1183 in GC2 for HABbaits1. A lower representation of ‘ancient’ sequences in inshore MCS3 is consistent with our observation of lower % eukaryote *sed*aDNA damage in sediments above ~ 35 cmbsf (i.e., the complete length of MCS3) (see section “Assessment of *sed*aDNA authenticity”. Damage profiles for *E. huxleyi sed*aDNA are much more variable in inshore MCS3 (and in the upper ~ 25 cmbsf of GC2; Figs. 5, 6; Supplementary Material Fig. 1, 2) than the profiles of deeper, more stable offshore GC2 samples, likely resulting from a scarcity of ancient reads in the upper sediment layers and *sed*aDNA damage patterns not being as pronounced as in deeper GC2 samples.

The *E. huxleyi sed*aDNA damage profiles of Shotgun, Planktonbaits1 and HABbaits1 showed similar variability with depth amongst each other (Figs. 5, 6; Supplementary Material Fig. 1, 2), suggesting that the hybridisation capture technique reliably preserves the DNA damage patterns of the original sample (represented by Shotgun) and is well-suited for the capture of past marine eukaryote *sed*aDNA. Further, HOPS provided a valid approach to authenticate *sed*aDNA from marine eukaryotes. We were unable to generate clear *sed*aDNA damage profiles from the upper ~ 25 to 35 cmbsf in both MCS3 (spanning the last ~ 145 years; Fig. 5; Supplementary Material Fig. 1) and GC2 (~ 1500 years, Fig. 6; Supplementary Material Fig. 2), indicating that *sed*aDNA damage is not as pronounced in the upper (younger) sediment layers at our study location and detectable only below that depth. Below ~ 35 cmbsf in GC2 the *E. huxleyi* DNA damage profiles assumed a typical U-shape as the number of mismatches at the end of DNA fragments increases (Fig. 6; Supplementary Material Fig. 2). Our *E. huxleyi sed*aDNA damage profiles are the first generated for a marine eukaryote—and extend over an ~ 8950-year timescale.

## Discussion

We applied two new RNA bait sets and hybridisation capture to inshore and offshore marine sediments to investigate marine eukaryotes more broadly (Planktonbaits1) and a more tailored approach focused on selected common and harmful taxa in our study region (HABbaits1). Our results show that hybridisation capture improves the yield of target eukaryote *sed*aDNA, and preserves DNA damage patterns, allowing us to assess *sed*aDNA authenticity, and generate the first ancient DNA damage profiles of a keystone marine phytoplankton organism, the ubiquitous coccolithophore *Emiliania huxleyi*.

Both Planktonbaits1 and HABbaits1 successfully captured *sed*aDNA of eukaryote organisms in two sediment cores collected off the Tasmanian east coast. Eukaryote *sed*aDNA has been repeatedly shown to be present in low amounts in seafloor sediments, limiting the metagenomic analysis and detailed reconstruction of past marine ecosystems. While both Planktonbaits1 and HABbaits1 achieved a considerable enrichment in Eukaryota for our inshore site MCS3 (4- to 9-fold, respectively), this increase was about half at the offshore site GC2. This difference may be due to the initial difference in proportions of Eukaryota DNA at the two sites. Shotgun showed Eukaryota contributed ~ 5% to the total pool of *sed*aDNA at MCS3, while contributing ~ 28% at GC2. The latter high proportion is primarily a result of a sharp increase in the relative abundance of Eukaryota in GC2 below 35 cmbsf. This initially relatively high proportion of Eukaryota sequences in GC2 *sed*aDNA extracts may have saturated the baits in our hybridisation reaction and would explain the less pronounced increase in GC2 Eukaryota proportions using either bait set. To further increase the Eukaryota signal in future studies, it may be beneficial to add a larger volume of baits (> 3 µL) to *sed*aDNA extracts expected (e.g., from shallow shotgun sequencing prior to enriching) to have a relatively high Eukaryota *sed*aDNA content.

The HOPS bioinformatic tool^23^ proved highly valuable in identifying and analysing ancient eukaryote sequences in our *sed*aDNA. The HOPS generated output of our ‘Eukaryota’ (taxa list *a*) run enabled the determination of ‘% eukaryote *sed*aDNA damage’, a parameter that can be used as a proxy of *sed*aDNA authenticity in the future. Here, the % eukaryote *sed*aDNA damage was in the lower quarter (1–24%), with a relatively high proportion of reads passing the default filtering criteria. The latter criteria used a minimum percent identity (mpi) level of 95%, a relatively stringent cut-off while still retaining the majority of reads. Lowering the mpi cut-off may have resulted in higher % eukaryote *sed*aDNA damage due to more reads passing the filtering criteria, however, this would also result in increased inaccuracies in taxa-assignments. It might be that ~ 24% comprises the maximum possible degree of eukaryote *sed*aDNA damage, for example, due to chromatin structures that may protect the majority of DNA regions while exposing others to degradation^32–34^. The latter might also explain the plateauing of the % eukaryote *sed*aDNA damage below ~ 30 cmbsf in GC2, however, this speculative and requires further investigation.

At both our inshore and offshore site, we observed a significant increase in % eukaryote *sed*aDNA damage with subseafloor depth, demonstrating that eukaryote *sed*aDNA shows increased DNA damage with increasing age of sediments. However, at both sites the *sed*aDNA damage was consistently lower in the upper 25–35 cmbsf (< 7% and < 15% at MCS3 and GC2, respectively), then increased sharply down-core from this depth (to ~ 6 to 11% and ~ 22% in MCS3 and GC2, respectively), and remained at this level towards the bottom of in GC2. At sites MCS3 and GC2, subseafloor depths of ~ 30 cmbsf correspond to sediment ages of ~ 124 and ~ 1271 years, respectively, which explains the much higher % eukaryote *sed*aDNA damage in GC2 (~ 22%) relative to MCS3 (< 10%) at this depth. It is possible that physical and/or chemical factors contribute to the increased preservation of *sed*aDNA damage patterns at depth, such as increased sediment compaction associated with less pore water movement and less oxygen exposure, as well as microbial activity^35–37^, and that the latter factors might play a greater role below 30 cmbsf at our sites. However, the above points on potential physico-chemical contributing factors to the variability in % eukaryote *sed*aDNA damage with subseafloor depth are speculative. Further research is required to determine whether reaching a ‘critical depth’ at which the % eukaryote *sed*aDNA damage first accelerates and the plateaus is a pattern characteristic of our study location, or of wider importance. If the latter holds true, then this ‘critical depth’ could be used as a guide to define ‘recent’ versus ‘ancient’ sediments, a distinction that has remained unclear in the *sed*aDNA research field to date. Future *sed*aDNA studies should also investigate how the % eukaryote *sed*aDNA damage varies in much older sediment records (older than Holocene) and depending on sediment properties (e.g., clay-rich sediments that appear to benefit DNA preservation^38^).

The strong positive correlation between % eukaryote *sed*aDNA damage amongst Shotgun, Planktonbaits1 and HABbaits1, demonstrates that DNA damage signals present in *sed*aDNA are preserved throughout the hybridisation capture approach. This is important as it allows the authentication of *sed*aDNA using bioinformatic tools, which any ancient DNA study should incorporate^23^. Through hybridisation capture more target sequences are available as input for DNA damage analysis software such as HOPS, which increases the robustness of such analyses^23^, thus is strongly recommended for *sed*aDNA analyses. While future refinement of the ‘% eukaryote *sed*aDNA damage’ measure may be necessary, our analyses show that it can be used as a proxy for *sed*aDNA authenticity in sediment records. Generally, for marine *sed*aDNA investigations of eukaryote taxa, the capacity to assess DNA damage provides a crucial advantage over metabarcoding where deamination patterns are removed throughout the amplification process and thus prevent damage-based authenticity assessments.

Running our data through the HOPS pipeline (taxa list *b*) and MEx-IPA allowed us to generate DNA damage plots for a key marine phytoplankton species, *Emiliania huxleyi*. This ubiquitous calcareous nanoplankton has thrived in the oceans since the Cretaceous, is one of the most abundant phytoplankton species in the global ocean and is ubiquitous from tropical to temperate to Antarctic Australian waters^39,40^. Consistent with its biogeographic distribution in the modern ocean, we expected to detect traces of this species in our *sed*aDNA, and in higher relative abundances offshore^39,40^.

We retained the maximum number of reads throughout our analyses (by examining proportions rather than rarefying our data), which enabled us to generate *E. huxleyi* DNA damage profiles from all three datasets, Shotgun, Planktonbaits1 and HABbaits1. The damage profiles generated by Shotgun, Planktonbaits1 and HABbaits1 for the same sample were very similar, indicating preservation of DNA damage patterns in our original sample (Shotgun) and in our enriched samples after hybridisation capture. Consistent with our finding of low eukaryote-derived *sed*aDNA damage in the upper 25–30 cmbsf, no clear *E. huxleyi* damage patterns could be determined from these depths. *sed*aDNA damage patterns with a typical U-shape were found only below ~ 25 cmbsf in GC2, again suggesting the existence of a critical depth below which DNA degradation becomes more pronounced, reinforcing the importance of investigating whether this phenomenon is of wider importance, and possibly influenced by physical or chemical properties, sedimentation rates, and/or microbial activity.

The study of marine *sed*aDNA offers huge potential for the comprehensive reconstruction of past marine ecosystems (including viruses, archaea, prokaryotes and eukaryotes). Eukaryotes (phytoplankton and higher organisms) are particularly popular study organisms due to their importance as primary producers and use as environmental indicators. However, *sed*aDNA studies focussing on eukaryotes have been severely limited by the very low amounts of DNA preserved in the subseafloor, and the lack of bioinformatic tools to authenticate these miniscule amounts of eukaryote *sed*aDNA in metagenomic data. To date, no marine *sed*aDNA studies have included authentication of *sed*aDNA (i.e., that the DNA recovered is ancient and not substantially impacted by contamination with modern DNA) a routine procedure for ancient DNA studies focussing on humans and megafauna. Our study provides a key advance in that we *(1)* used a hybridisation capture technique to enrich target marine eukaryote *sed*aDNA independent of DNA fragment size, and *(2)* applied the recently developed bioinformatic tool HOPS for *sed*aDNA damage analysis and to bioinformatically authenticate our marine *sed*aDNA. These advances provide a critical benefit to studies of paleo-community composition from *sed*aDNA, and the detailed investigation of both model and non-model organisms within these communities.

## Conclusions

In this study we show the reliability of the hybridisation capture as a novel tool for investigating changing patterns of abundance of marine eukaryotes from their *sed*aDNA in seafloor sediments. We furthermore applied a recently developed bioinformatic tool for metagenomic DNA damage analysis (HOPS) to our *sed*aDNA, which allowed us to develop a new measure for *sed*aDNA authenticity (‘% eukaryote *sed*aDNA damage’) that changes with subseafloor depth. Through our *sed*aDNA damage analysis were also able to generate *sed*aDNA damage profiles of the ubiquitous coccolithophore *E. huxleyi*, the first ever such profiles generated for a marine eukaryote—extending over an 8000-year timescale. Our study provides a major step forward for the future investigation of eukaryotes from marine *sed*aDNA, enabling detailed insights into past marine ecosystem composition over geological timescales.

## Supplementary Information

Supplementary Information.

## Data Availability

The demultiplexed raw sequencing data analysed during this study are not yet publicly available due to ongoing manuscript preparations but are available from the corresponding author upon reasonable request. Once publications are finalised, the data will be made openly available via the NCBI Sequence Read Archive (https://www.ncbi.nlm.nih.gov/sra).

## References

[1] Szczuciński W (2016). Ancient sedimentary DNA reveals past tsunami deposits. Mar. Geol..

[2] More KD (2018). A 43 kyr record of protist communities and their response to oxygen minimum zone variability in the Northeastern Arabian Sea. Earth Planet. Sci. Lett..

[3] De Schepper S (2019). The potential of sedimentary ancient DNA for reconstructing past sea ice evolution. ISME J..

[4] Armbrecht LH (2019). Ancient DNA from marine sediments: Precautions and considerations for seafloor coring, sample handling and data generation. Earth-Sci. Rev..

[5] Verity PG, Smetacek V (1996). Organism life cycles, predation, and the structure of marine pelagic ecosystems. Mar. Ecol. Prog. Ser..

[6] Hays GC, Richardson AJ, Robinson C (2005). Climate change and marine plankton. Trends Ecol. Evol..

[7] Armbrecht L (2020). An optimized method for the extraction of ancient eukaryote DNA from marine sediments. Mol. Ecol. Resour..

[8] Armbrecht L (2020). The potential of sedimentary ancient DNA to reconstruct past ocean ecosystems. Oceanography.

[9] Taberlet P, Coissac E, Pompanon F, Brochmann C, Willerslev E (2012). Towards next-generation biodiversity assessment using DNA metabarcoding. Mol. Ecol..

[10] Pedersen MW (2015). Ancient and modern environmental DNA. Philos. Trans. R Soc. B. Biol. Sci..

[11] Weyrich LS (2017). Neanderthal behaviour, diet, and disease inferred from ancient DNA in dental calculus. Nature.

[12] Horn S, Shapiro B, Hofreiter M (2012). Target enrichment via DNA hybridisation capture. Ancient DNA, Methods and Protocols.

[13] Foster NR, Gillanders BM, Jones AR, Young JM, Waycott M (2020). A muddy time capsule: using sediment environmental DNA for the long-term monitoring of coastal vegetated ecosystems. Mar. Freshw. Res..

[14] Paijmans JLA, Gilbert MTP, Hofreiter M (2013). Mitogenomic analyses from ancient DNA. Mol. Phylogenet. Evol..

[15] Li C (2015). DNA capture reveals transoceanic gene flow in endangered river sharks. Proc. Natl. Acad. Sci. USA.

[16] Murchie T (2019). PalaeoChip Arctic1. 0: An optimised eDNA targeted enrichment approach to reconstructing past environments. bioRxiv.

[17] MyBaits Manual v.4.01—Hybridization Capture for Targeted NGS, 2018. https://arborbiosci.com/wp-content/uploads/2019/08/myBaits-Manual-v4.pdf.

[18] Ginolhac A, Rasmussen M, Gilbert MTP, Willerslev E, Orlando L (2011). mapDamage: testing for damage patterns in ancient DNA sequences. Bioinformatics.

[19] Jónsson H, Ginolhac A, Schubert M, Johnson PLF, Orlando L (2013). MapDamage2.0: fast approximate Bayesian estimates of ancient DNA damage parameters. Bioinformatics.

[20] Llamas B (2015). Late Pleistocene Australian marsupial DNA clarifies the affinities of extinct megafaunal kangaroos and wallabies. Mol. Biol. Evol..

[21] Tobler R (2017). Aboriginal mitogenomes reveal 50,000 years of regionalism in Australia. Nature.

[22] Collin TC (2020). An open-sourced bioinformatic pipeline for the processing of Next-Generation Sequencing derived nucleotide reads: Identification and authentication of ancient metagenomic DNA. bioRxiv.

[23] Hübler R (2019). HOPS: automated detection and authentication of pathogen DNA in archaeological remains. Genome Biol..

[24] Willerslev E, Cooper A (2005). Ancient DNA. Proc. R. Soc. B Biol. Sci..

[25] De Vargas, C. *et al.* Eukaryotic plankton diversity in the sunlit ocean. *Science* **348**, 1261605–1/11 (2015). 10.1126/science.1261605 25999516

[26] Quast C (2013). The SILVA ribosomal RNA gene database project: Improved data processing and web-based tools. Nucleic Acids Res..

[27] Guillou L (2013). The Protist Ribosomal Reference database (PR2): A catalog of unicellular eukaryote Small Sub-Unit rRNA sequences with curated taxonomy. Nucleic Acids Res..

[28] Grzebyk, D., Audic, S., Decelle, J. & de Vargas, C. PHYTOPK28-D1D2: A curated database of 28S rRNA gene D1–D2 domains from eukaryotic organisms dedicated to metabarcoding analyses of marine phytoplankton samples. *Mendeley Data* **1**. 10.17632/mndb4h87yg.1 (2017).

[29] Ratnasingham S, Hebert PDN (2007). The Barcode of life data system. Mol. Ecol. Notes.

[30] Meyer M, Kircher M (2010). Illumina sequencing library preparation for highly multiplexed target capture and sequencing. Cold Spring Harb. Protoc..

[31] Hammer Ø, Harper DAT, Ryan PD (2001). PAST: Paleontological Statistics software package for education and data analysis. Palaeontol. Electron..

[32] Takata H (2013). Chromatin compaction protects genomic DNA from radiation damage. PLoS ONE.

[33] Kistler L, Ware R, Smith O, Collins M, Allaby RG (2017). A new model for ancient DNA decay based on paleogenomic meta-analysis. Nucleic Acids Res..

[34] Gornik SG, Hu I, Lassadi I, Waller RF (2019). The biochemistry and evolution of the dinoflagellate nucleus. Microorganisms.

[35] Eglinton G, Logan GA (1991). Molecular preservation. Philos. Trans. R. Soc. London B.

[36] Palchevskiy V, Finkel SE (2006). Escherichia coli competence gene homologs are essential for competitive fitness and the use of DNA as a nutrient. J. Bacteriol..

[37] Corinaldesi C, Beolchini F, Dell’Anno A (2008). Damage and degradation rates of extracellular DNA in marine sediments: Implications for the preservation of gene sequences. Mol. Ecol..

[38] Vuillemin A (2019). Archaea dominate oxic subseafloor communities over multimillion-year time scales. Sci. Adv..

[39] Hallegraeff GM (1984). Coccolithophorids (calcareous nanoplankton) from Australian waters. Bot. Mar..

[40] Cubillos JC (2007). Calcification morphotypes of the coccolithophorid Emiliania huxleyi in the Southern Ocean: Changes in 2001 to 2006 compared to historical data. Mar. Ecol. Prog. Ser..

