# Supplementary material for: Hybridisation capture allows DNA damage analysis of ancient marine eukaryotes
Source: Sci Rep. 2021 Feb 5;11:3220. doi: 10.1038/s41598-021-82578-6 (PMC7864908; doi:10.1038/s41598-021-82578-6)
Supplement: Supplementary file 1 — Supplementary Information. [file 41598_2021_82578_MOESM1_ESM.docx]

**Supplementary Material**

**Hybridisation capture allows DNA damage analysis of ancient marine eukaryotes**

**Armbrecht, L.**^*^, **Hallegraeff, G., Bolch, C.J.S., Woodward, C., Cooper, A.**

*Correspondence: Linda Armbrecht, Email: linda.armbrecht@adelaide.edu.au

**Contents Page**

## Methods - SedaDNA extractions 2

## Methods - Metagenomic shotgun library preparation 3

Methods - *Bioinformatics: Raw sequencing data processing* 4

References 5

Supplementary Material Figures - *Supplementary Material Figure 1* 7

Supplementary Material Figures - *Supplementary Material Figure 2* 9

Supplementary Material Tables - *Supplementary Material Table 1* 12

Supplementary Material Tables - *Supplementary Material Table 2*  18

## Supplementary Material Tables - Supplementary Material Table 3 33

**Methods**

## SedaDNA extractions

Our extraction method followed the optimised (“combined”) approach described previously^1^. In brief, we used 0.25 g of each sample and incubated in 0.75 mL EDTA overnight on a rotary mixer (room temperature, RT). After centrifuging (3 min, 13,000 rpm), we kept the supernatant (RT) while proceeding with bead-beating of the pellet (3 x 20 s with 5 s breaks) in 0.75 mL customary beat-beating and C1 solution (DNeasy PowerLyzer PowerSoil Kit, Qiagen) using a Precellys 24 homogenizer (Bertin Instruments, France) and a FastPrep FP120 (Thermo Savant, USA) for the MCS3 and GC2 samples, respectively, followed by centrifugation (3 min, 10,319 rpm). We combined purified DNA-solutions from EDTA and bead-beating at 0.75 mL each per sample, added this DNA to 6 mL modified QG binding buffer (Qiagen) with 100 µL liquid silica^2^ in a 15 mL centrifugation tube, and stirred the solution on a rotary mixer (1 hr, RT). After centrifuging (1 min, 14,000 rpm), we resuspended and washed the pellet with 0.9 mL of QG binding buffer, and twice with 80% EtOH with each wash step followed by centrifugation (1 min, 14,000 rpm). DNA pellets were then dried (15 min, 37 °C) and resuspended in 100 µL TLE Buffer (50 μL Tris HCL (1M), 10 μL EDTA (0.5M), 5 mL nuclease-free water). Following incubation (10 min, 50 °C), we centrifuged (1 min, 14,000 rpm) and stored the supernatant (free of silica) in a sterile Lo-bind tube (Eppendorf) at -20 °C. To monitor laboratory contamination, we used extraction blank controls (EBCs) by processing 1-2 (depending on the extraction-batch size) empty bead-tubes through the extraction protocol. A total of 30 extracts were generated from sediment samples and 7 extracts from EBCs.

## Metagenomic shotgun sequencing library preparations

We followed the shotgun sequencing library preparation protocol detailed previously^1^, with the following modifications. We used 3 µL of the Bst-reaction product as input for a 25 µL PCR with the primers IS7 and IS8^3^ (preparing 5 PCR replicates for most samples; however, for twelve offshore core samples (and two associated EBCs) we ran 8 PCR replicates, and for three MCS3 samples (0 - 1.5 cm, 12 - 13.5 cm, 34 - 35.5cm) and the associated EBC we only ran duplicates; with the number of PCR replicates varying due to DNA template limitations resulting from previous PCR trials). Each PCR reaction included 14.2 nuclease-free H_2_O, 2.5 µL 10× Gold Buffer, 2.5 µL 25mM MgCl_2_, 0.25 µL 25mM dNTPs, 1.25 IS7, 1.25 IS8, and 0.1 µL AmpliTaq Gold Polymerase (Applied Biosystems). Thermal cycling specifications were as follows: 6 min at 94 °C, 18 cycles (22 cycles in the case of the 12 GC2 samples and the two associated EBCs) of 30 s denaturation at 94 °C, 30 s annealing at 60 °C, 40 s extension at 72 °C, and 10 min of final extension. We purified our PCR products using AxyPrep magnetic beads (Axygen Biosciences; 1:1.8 library:beads), eluted the DNA in Buffer EB (Qiagen) with 0.05% Tween20 (Sigma Aldrich), and quantified DNA concentrations using Qubit dsDNA HS Assays (Molecular Probes). Next, we ran additional PCRs (25 µL reactions, 5 replicates each) using the same reagents as above but with the Indexing Primer IS4, a GAII index^3^ and 13 cycles. We pooled the PCR products per sample, cleaned them using AxyPrep magnetic beads (1:1.1 library:beads), and performed quantity and quality checks through TapeStation (Agilent Technologies, USA). For those samples showing primer-dimer, we repeated the AxyPrep clean-up (1:1.1 library:beads) and TapeStation control. We prepared two equimolar (7.3 nM and 10 nM as per TapeStation) sequencing pools, to which we added the libraries prepared from EBCs in a 1 in 10 dilution. We submitted the final pools for Illumina NextSeq sequencing (2 × 75 bp cycle) at the Australian Cancer Research Foundation Cancer Genomics Facility & Centre for Cancer Biology, Adelaide, Australia, and at the Garvan Institute of Medical Research, KCCG Sequencing Laboratory (Kinghorn Centre for Clinical Genomics) Darlinghurst, Australia.

### Bioinformatics

*Raw sequencing data processing*

Bioinformatic processing and filtering of the sequencing data, hereafter referred to as datasets “Shotgun”, “Planktonbaits1” and “HABbaits1”, followed established protocols^1^. We ran FastQC and MultiQC quality controls on the raw sequencing data and after each step of data filtering (FastQC v0.11.8, Babraham Bioinformatics; MultiQC^4^ v1.0.dev0. Demultiplexing and adapter trimming was performed using AdapterRemoval^5^ v2.3.0), which included the removal of consecutive stretches of low-quality bp and N’s, allowing for a barcode mismatch of 1 bp, discarding reads <25 bp and merging (collapsing) reads into .gz output files. We removed low-complexity sequences using the software Komplexity^6^ (--threshold 0.55) and removed duplicate sequences using the dedupe tool in BBMap v37.36. To retain the maximum number of reads (especially important for DNA damage assessment), we avoided subsampling (rarefying) our data and used relative abundances in the downstream analyses. We used the NCBI Nucleotide database (ftp://ftp.ncbi.nlm.nih.gov/blast/db/FASTA/nt.gz, downloaded November, 2019) as the reference database to build a MALT index (Step 3) and aligned our sequences using MALT^7^ (version 0.4.0; semiglobal alignment). The resulting .blastn files were converted to .rma6 format using the Blast2RMA tool in MEGAN6^8^ (version 6_18_9) with the default settings except for a minimum support percent of zero (‘off’) and a minimum percent identity of 95%. Subtractive filtering (i.e., subtracting reads for species identified in EBCs from samples) was conducted for each dataset separately^1^; hereafter, the term ‘samples’ refers to sediment-derived data post-EBC subtraction). Computer code has been provided previously^1^ with the updated program versions used in this publication as per this section above.

**References**

1. Armbrecht, L. *et al.* An optimized method for the extraction of ancient eukaryote DNA from marine sediments. *Mol. Ecol. Resour.* **20**, 906–919 (2020).

2. Brotherton, P. *et al.* Neolithic mitochondrial haplogroup H genomes and the genetic origins of Europeans. *Nat. Commun.* **4**, 1711–1719 (2013).

3. Meyer, M. & Kircher, M. Illumina sequencing library preparation for highly multiplexed target capture and sequencing. *Cold Spring Harb. Protoc.* **5**, (2010).

4. Ewels, P., Magnusson, M., Lundin, S. & Käller, M. MultiQC: Summarize analysis results for multiple tools and samples in a single report. *Bioinformatics* **32**, 3047–3048 (2016).

5. Schubert, M., Lindgreen, S. & Orlando, L. AdapterRemoval v2: Rapid adapter trimming, identification, and read merging. *BMC Res. Notes* **9**, 88 (2016).

6. Clarke, E. L. *et al.* Sunbeam: An extensible pipeline for analyzing metagenomic sequencing experiments. *Microbiome* **7**, 46 (2019).

7. Herbig, A. *et al.* MALT: Fast alignment and analysis of metagenomic DNA sequence data applied to the Tyrolean Iceman. *bioRxiv* (2016) doi:10.1101/050559.

8. Huson, D. H. *et al.* MEGAN Community Edition - Interactive Exploration and Analysis of Large-Scale Microbiome Sequencing Data. *PLoS Comput. Biol.* **12**, e1004957 (2016).

9. Hübler, R. *et al.* HOPS: automated detection and authentication of pathogen DNA in archaeological remains. *Genome Biol.* **20**, 1–13 (2019).

**Supplementary Material Figures:**

**
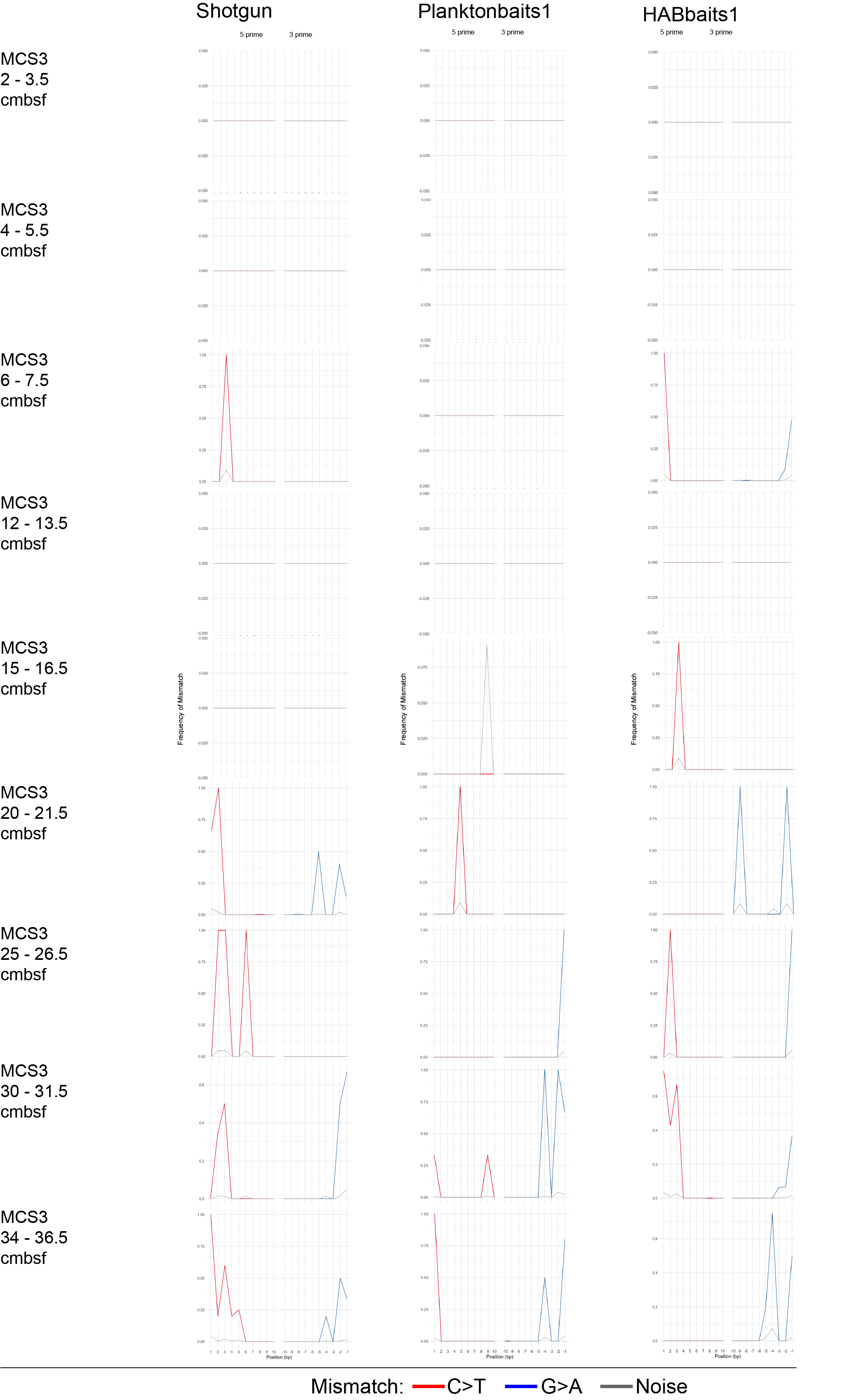
**

**Supplementary Material Figure 1: *Sed*aDNA damage profiles of *Emiliania huxleyi* in MCS3*.***  *E. huxleyi sed*aDNA damage profiles (frequency of mismatch against base pair position) per sample for Shotgun, Planktonbaits1 and HABbaits1 in MCS3. The red and blue lines denote C>T substitutions in 5' direction and G>A substitutions in 3’ direction, respectively, for all ancient alignments. Grey lines denote estimated noise^9^.

**
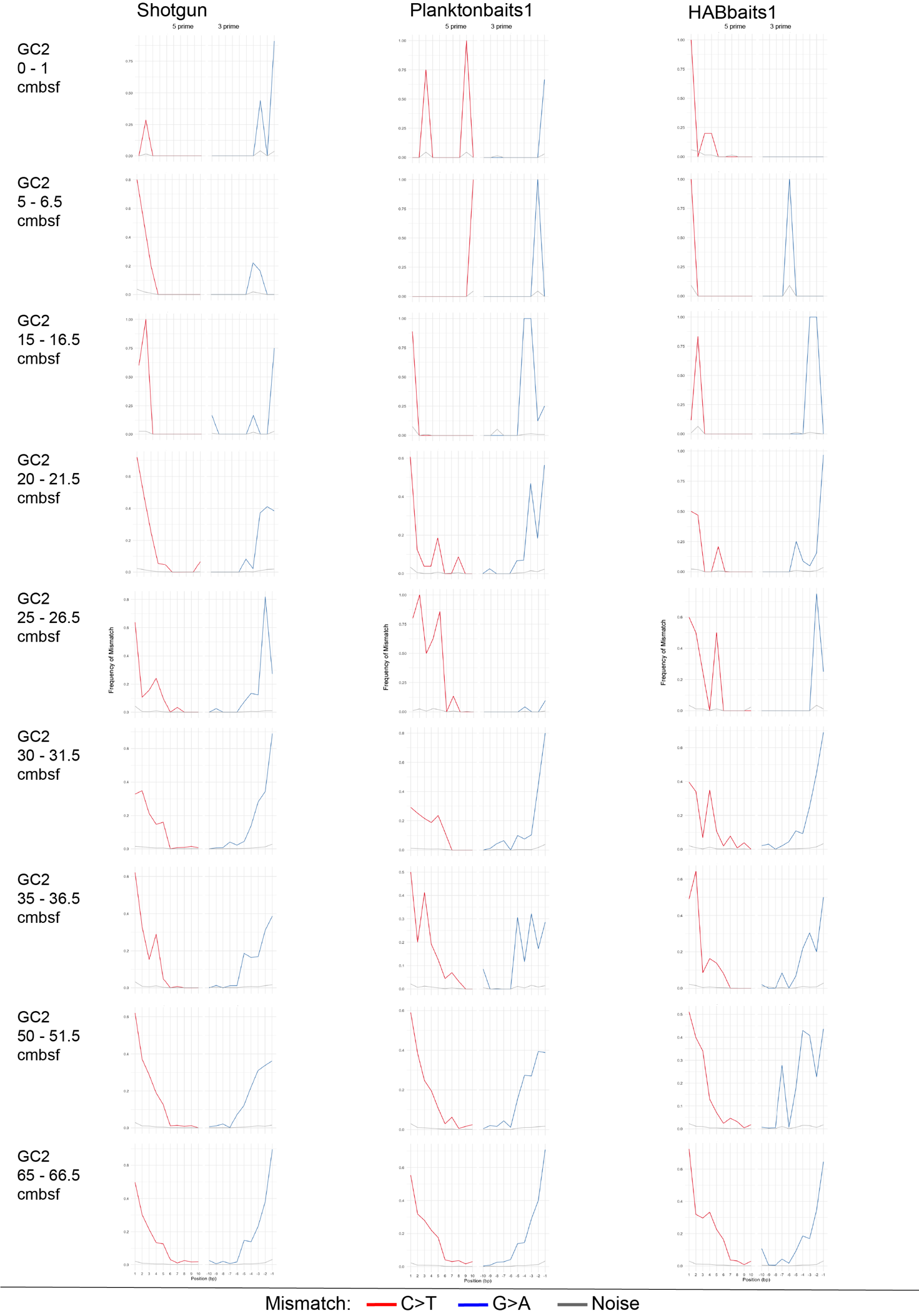
**

**Supplementary Material Figure 2: *Sed*aDNA damage profiles of *Emiliania huxleyi* in GC2*.***  *E. huxleyi sed*aDNA damage profiles (frequency of mismatch against base pair position) per sample for Shotgun, Planktonbaits1 and HABbaits1 in GC2 (listed from top-down with GC2 profiles continuing in the second column for each dataset). The red and blue lines denote C>T substitutions in 5' direction and G>A substitutions in 3’ direction, respectively, for all ancient alignments. Grey lines denote estimated noise^9^.

**
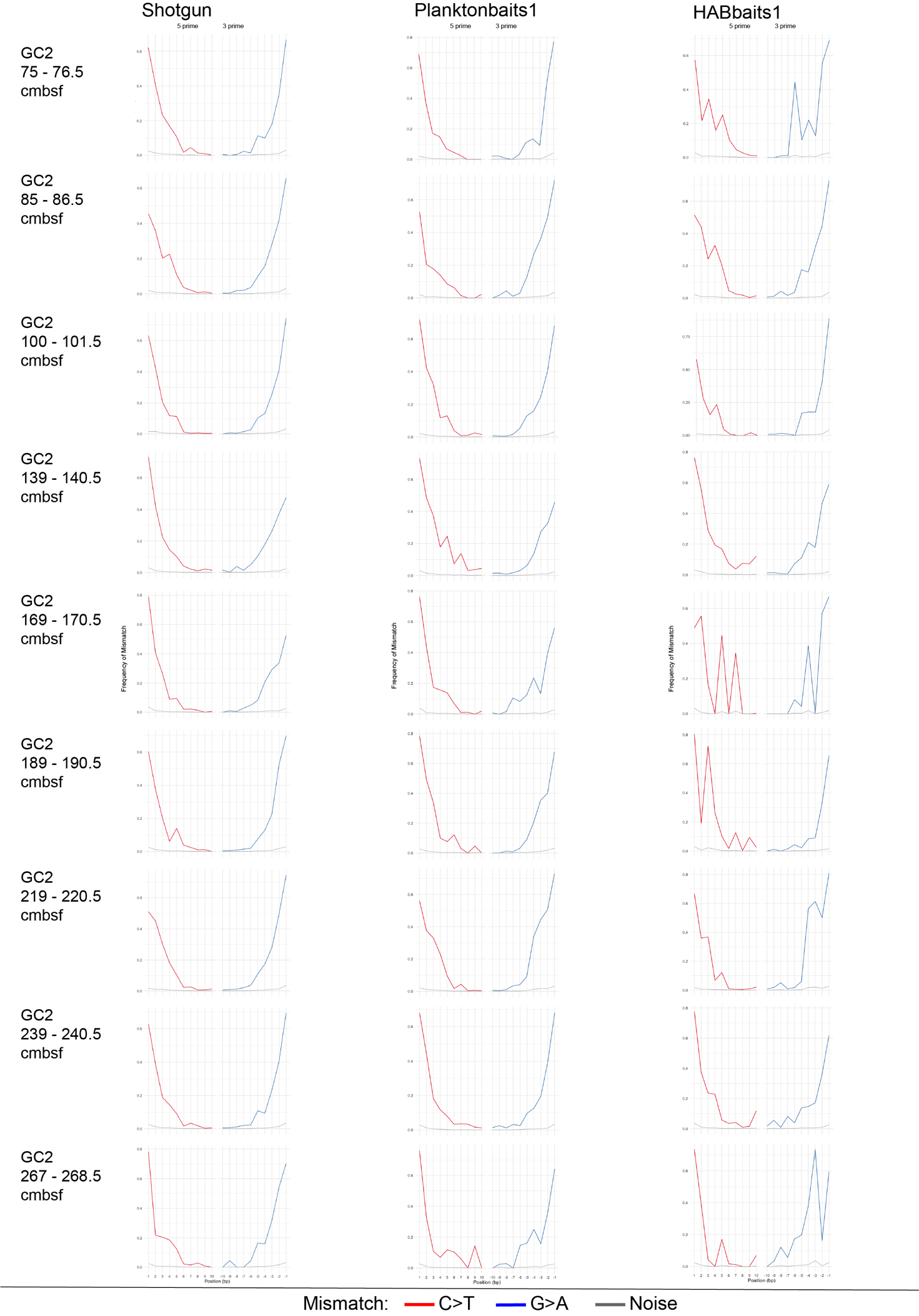
**

**Supplementary Material Figure 2 (continued): *Sed*aDNA damage profiles of *Emiliania huxleyi* in GC2*.***  *E. huxleyi sed*aDNA damage profiles (frequency of mismatch against base pair position) per sample for Shotgun, Planktonbaits1 and HABbaits1 in GC2 (listed from top-down with GC2 profiles continuing in the second column for each dataset). The red and blue lines denote C>T substitutions in 5' direction and G>A substitutions in 3’ direction, respectively, for all ancient alignments. Grey lines denote estimated noise^9^.

**Supplementary Material Tables**

**Supplementary Material Table 1.** Eukaryote taxa determined in extraction blank controls (EBCs) of Shotgun, Planktonbaits1 and HABbaits1 and removed from downstream analyses. For Shotgun we determined <0.7Mio and <0.5Mio reads in EBCs pre- and post-filtering, and for Planktonbaits1 <0.3 and <0.1, as per MultiQC. For HABbaits1 the number of reads detected in EBCs was too small for MultiQC reporting (“0Mio”). The total number of eukaryote reads in EBCs of Shotgun, Planktonbaits1 and HABbaits1 was 3,211, 1,179, and 128, respectively.

| **Shotgun** | **SG_21980A_EBC** | **SG_22301A_EBC** | **SG_22302A_EBC** | **SG_23351A_EBC** | **SG_23352A_EBC** | **SG_24029A_EBC** | **SG_24030A_EBC** |
| --- | --- | --- | --- | --- | --- | --- | --- |
| *Plasmodium vivax* | 0 | 0 | 0 | 0 | 2 | 0 | 0 |
| *Plasmodium berghei* | 0 | 0 | 0 | 0 | 0 | 1 | 0 |
| *Eimeria acervulina* | 0 | 1 | 3 | 3 | 0 | 0 | 0 |
| *Stenamoeba* | 0 | 0 | 0 | 1 | 0 | 0 | 0 |
| *Venturia effusa* | 0 | 0 | 0 | 1 | 0 | 0 | 0 |
| *Aureobasidium pullulans* | 0 | 0 | 0 | 1 | 0 | 0 | 0 |
| *Alternaria alternata* | 0 | 0 | 0 | 2 | 0 | 0 | 0 |
| *Alternaria arborescens* | 0 | 0 | 0 | 1 | 0 | 0 | 0 |
| *Penicillium chrysogenum* | 0 | 0 | 0 | 1 | 0 | 0 | 0 |
| *Penicillium expansum* | 0 | 0 | 0 | 1 | 0 | 0 | 0 |
| *Thelebolaceae* | 0 | 0 | 0 | 1 | 0 | 0 | 0 |
| *Beauveria bassiana* | 0 | 0 | 0 | 0 | 1 | 0 | 0 |
| *Cordyceps militaris* | 0 | 1 | 0 | 0 | 3 | 0 | 0 |
| *Lecanicillium* | 0 | 0 | 0 | 0 | 3 | 0 | 0 |
| *Trichoderma* | 0 | 2 | 0 | 0 | 0 | 0 | 0 |
| *Fusarium fujikuroi* | 0 | 0 | 0 | 1 | 0 | 0 | 0 |
| *Fusarium verticillioides* | 0 | 0 | 0 | 7 | 0 | 0 | 0 |
| *Fusarium oxysporum* | 1 | 0 | 0 | 0 | 0 | 0 | 0 |
| *Fusarium graminearum* | 0 | 0 | 0 | 2 | 0 | 0 | 0 |
| *Fusarium pseudograminearum* | 0 | 0 | 0 | 1 | 0 | 0 | 0 |
| *Chaetomium globosum* | 0 | 0 | 0 | 1 | 0 | 0 | 0 |
| *Phaeoacremonium minimum* | 0 | 0 | 0 | 2 | 0 | 0 | 0 |
| *Debaryomyces hansenii* | 2 | 3 | 0 | 0 | 1 | 0 | 0 |
| *Saccharomyces cerevisiae* | 0 | 1 | 0 | 0 | 0 | 0 | 0 |
| *Trametes versicolor* | 0 | 0 | 0 | 2 | 0 | 0 | 0 |
| *Stereum hirsutum* | 0 | 0 | 0 | 12 | 0 | 0 | 0 |
| *Xylobolus sp. 101 OA-2013* | 0 | 0 | 0 | 2 | 0 | 0 | 0 |
| *Filobasidium uniguttulatum* | 0 | 0 | 0 | 1 | 0 | 0 | 0 |
| *Cryptococcus amylolentus* | 0 | 0 | 0 | 1 | 0 | 0 | 0 |
| *Kockovaella* | 0 | 0 | 0 | 1 | 0 | 0 | 0 |
| *Trichosporon asahii* | 1 | 0 | 0 | 1 | 0 | 0 | 0 |
| *Sporidiobolaceae* | 0 | 0 | 0 | 0 | 0 | 0 | 1 |
| *Sporisorium graminicola* | 0 | 0 | 0 | 1 | 0 | 0 | 0 |
| *Fungi incertae sedis* | 0 | 0 | 0 | 1 | 0 | 0 | 0 |
| *Larimichthys crocea* | 1 | 5 | 0 | 0 | 1 | 0 | 0 |
| *Salarias fasciatus* | 0 | 1 | 0 | 0 | 0 | 2 | 0 |
| *Oncorhynchus nerka* | 0 | 0 | 0 | 0 | 1 | 0 | 0 |
| *Oncorhynchus tshawytscha* | 0 | 0 | 0 | 29 | 0 | 0 | 0 |
| *Clupea harengus* | 0 | 2 | 0 | 0 | 0 | 0 | 0 |
| *Cyprinus carpio* | 25 | 570 | 10 | 41 | 127 | 123 | 27 |
| *Sinocyclocheilus anshuiensis* | 0 | 1 | 0 | 0 | 0 | 0 | 0 |
| *Heterocephalus glaber* | 22 | 5 | 3 | 5 | 1 | 1 | 0 |
| *Ictidomys tridecemlineatus* | 315 | 243 | 38 | 4 | 192 | 51 | 4 |
| *Macaca fascicularis* | 0 | 1 | 0 | 0 | 0 | 0 | 0 |
| *Homo sapiens* | 9 | 17 | 0 | 9 | 21 | 0 | 126 |
| *Pan troglodytes* | 0 | 0 | 0 | 1 | 0 | 0 | 3 |
| *Pongo abelii* | 0 | 15 | 0 | 0 | 0 | 14 | 0 |
| *Aotus nancymaae* | 0 | 0 | 0 | 0 | 0 | 0 | 1 |
| *Panthera pardus* | 0 | 1 | 0 | 0 | 0 | 1 | 0 |
| *Bos mutus* | 0 | 4 | 0 | 0 | 1 | 0 | 0 |
| *Bos taurus* | 3 | 0 | 0 | 0 | 0 | 0 | 0 |
| *Capra hircus* | 0 | 0 | 0 | 1 | 0 | 0 | 0 |
| *Ovis canadensis* | 0 | 1 | 0 | 0 | 0 | 0 | 0 |
| *Odocoileus hemionus* | 0 | 1 | 0 | 0 | 0 | 0 | 0 |
| *Odocoileus virginianus* | 0 | 1 | 0 | 0 | 0 | 0 | 0 |
| *Camelus ferus* | 13 | 7 | 0 | 6 | 0 | 8 | 0 |
| *Aquila chrysaetos* | 0 | 0 | 0 | 33 | 0 | 0 | 0 |
| *Columba livia* | 0 | 0 | 0 | 19 | 0 | 0 | 0 |
| *Streptopelia turtur* | 0 | 0 | 0 | 115 | 0 | 0 | 0 |
| *Falco peregrinus* | 0 | 0 | 0 | 1 | 0 | 0 | 0 |
| *Gallus gallus* | 0 | 0 | 0 | 1 | 0 | 0 | 0 |
| *Corvus brachyrhynchos* | 0 | 0 | 0 | 4 | 0 | 0 | 0 |
| *Cyanistes caeruleus* | 0 | 0 | 0 | 2 | 0 | 0 | 0 |
| *Parus major* | 0 | 0 | 0 | 1 | 0 | 0 | 0 |
| *Zonotrichia albicollis* | 0 | 0 | 0 | 1 | 0 | 0 | 0 |
| *Taeniopygia guttata* | 0 | 0 | 0 | 7 | 0 | 0 | 0 |
| *Serinus canaria* | 0 | 0 | 0 | 2 | 0 | 0 | 0 |
| *Sturnus vulgaris* | 0 | 0 | 0 | 34 | 0 | 0 | 0 |
| *Acrocephalus arundinaceus* | 0 | 0 | 0 | 1 | 0 | 0 | 0 |
| *Phaethon lepturus* | 0 | 0 | 0 | 1 | 0 | 0 | 0 |
| *Apteryx australis* | 0 | 4 | 0 | 1 | 0 | 0 | 0 |
| *Diphyllobothrium* | 0 | 325 | 0 | 3 | 2 | 35 | 0 |
| *Dicrocoelium dendriticum* | 0 | 8 | 0 | 58 | 0 | 2 | 0 |
| *Trichobilharzia regenti* | 0 | 0 | 0 | 0 | 1 | 0 | 0 |
| *Brugia timori* | 0 | 0 | 0 | 6 | 0 | 0 | 0 |
| *Strongyloides stercoralis* | 0 | 0 | 0 | 0 | 2 | 0 | 0 |
| *Strongyloides venezuelensis* | 0 | 0 | 0 | 1 | 0 | 0 | 0 |
| *Nippostrongylus brasiliensis* | 0 | 2 | 0 | 14 | 39 | 7 | 2 |
| *Parasteatoda tepidariorum* | 0 | 1 | 0 | 0 | 0 | 0 | 0 |
| *Moina brachiata* | 0 | 0 | 0 | 0 | 0 | 1 | 0 |
| *Hyalella azteca* | 0 | 0 | 0 | 2 | 0 | 0 | 0 |
| *Proasellus solanasi* | 0 | 3 | 0 | 1 | 0 | 4 | 0 |
| *Ostrinia furnacalis* | 0 | 0 | 0 | 1 | 0 | 0 | 0 |
| *Harmonia axyridis* | 0 | 0 | 0 | 2 | 0 | 0 | 0 |
| *Drosophila biarmipes* | 0 | 0 | 0 | 1 | 0 | 0 | 0 |
| *Drosophila pseudoobscura* | 0 | 2 | 0 | 0 | 0 | 3 | 0 |
| *Culex pipiens* | 0 | 0 | 0 | 1 | 0 | 0 | 0 |
| *Camponotus floridanus* | 0 | 8 | 0 | 0 | 0 | 0 | 0 |
| *Rhopalosiphum maidis* | 0 | 0 | 0 | 2 | 0 | 0 | 0 |
| *Psylloidea* | 0 | 0 | 0 | 1 | 0 | 0 | 0 |
| *Conus episcopatus* | 0 | 0 | 0 | 1 | 0 | 0 | 0 |
| *Acropora digitifera* | 0 | 25 | 5 | 0 | 0 | 0 | 0 |
| *Dendronephthya gigantea* | 0 | 0 | 0 | 1 | 0 | 0 | 0 |
| *Cercomonadida* | 0 | 0 | 0 | 1 | 0 | 0 | 0 |
| *Saprolegnia parasitica* | 0 | 0 | 0 | 1 | 0 | 0 | 0 |
| *Chlorococcum tatrense* | 0 | 0 | 0 | 4 | 0 | 0 | 0 |
| *Volvox carteri* | 0 | 0 | 0 | 1 | 0 | 0 | 0 |
| *Mychonastes homosphaera* | 0 | 0 | 0 | 2 | 0 | 0 | 0 |
| *Monoraphidium neglectum* | 0 | 0 | 0 | 1 | 0 | 0 | 0 |
| *Micractinium conductrix* | 0 | 0 | 0 | 1 | 0 | 0 | 0 |
| *Coccomyxa sp. SUA001* | 0 | 0 | 0 | 1 | 0 | 0 | 0 |
| *Physcomitrella patens* | 1 | 0 | 0 | 0 | 0 | 0 | 0 |
| *Dioon* | 9 | 38 | 0 | 3 | 0 | 89 | 0 |
| *Pinus taeda* | 0 | 0 | 0 | 8 | 0 | 0 | 0 |
| *Daucus carota* | 0 | 0 | 0 | 12 | 0 | 0 | 0 |
| *Lasthenia californica* | 22 | 0 | 0 | 0 | 0 | 0 | 0 |
| *Chionanthus rupicola* | 0 | 0 | 0 | 1 | 0 | 0 | 0 |
| *Physochlaina orientalis* | 0 | 0 | 0 | 1 | 0 | 0 | 0 |
| *Nepenthes ventricosa x N. alata* | 0 | 0 | 0 | 1 | 0 | 0 | 0 |
| *Phaseolus* | 0 | 1 | 0 | 0 | 0 | 0 | 0 |
| *Quercus suber* | 0 | 0 | 0 | 2 | 0 | 0 | 0 |
| *Populus trichocarpa* | 0 | 0 | 0 | 3 | 0 | 0 | 0 |
| *Pyrus x bretschneideri* | 0 | 0 | 0 | 0 | 0 | 0 | 1 |
| *Theobroma cacao* | 0 | 5 | 0 | 0 | 0 | 0 | 0 |
| *Rhodamnia argentea* | 0 | 1 | 0 | 0 | 0 | 0 | 0 |
| *Citrus sinensis* | 0 | 0 | 0 | 1 | 0 | 0 | 0 |
| *Elaeis guineensis* | 0 | 0 | 0 | 5 | 0 | 0 | 0 |
| *Oryza sativa* | 0 | 0 | 0 | 1 | 0 | 0 | 0 |
| *Triticum aestivum* | 0 | 1 | 0 | 0 | 0 | 0 | 0 |
| *Triticum monococcum* | 0 | 0 | 0 | 0 | 0 | 0 | 1 |
| *Zea mays* | 0 | 0 | 0 | 3 | 0 | 0 | 0 |
| **Planktonbaits1** | **HYB18SV9_A21980_EBC** | **HYB18SV9_A22301_EBC** | **HYB18SV9_A22302_EBC** | **HYB18SV9_A23351_EBC** | **HYB18SV9_A23352_EBC** | **HYB18SV9_A24029_EBC** | **HYB18SV9_A24030_EBC** |
| *Opegrapha vulgata* | 0 | 0 | 0 | 0 | 0 | 0 | 1 |
| *Aureobasidium pullulans* | 0 | 0 | 0 | 2 | 0 | 0 | 0 |
| *Alternaria solani* | 0 | 0 | 0 | 1 | 0 | 0 | 0 |
| *Penicillium* | 0 | 0 | 0 | 2 | 0 | 0 | 0 |
| *Metarhizium robertsii* | 0 | 0 | 0 | 0 | 0 | 0 | 1 |
| *Fusarium graminearum* | 0 | 0 | 0 | 1 | 0 | 0 | 0 |
| *Microascales* | 0 | 0 | 0 | 1 | 0 | 0 | 0 |
| *Saccharomyces cerevisiae* | 0 | 1 | 0 | 0 | 0 | 0 | 0 |
| *Stereum hirsutum* | 0 | 0 | 0 | 5 | 0 | 0 | 0 |
| *Cryptococcus neoformans* | 0 | 0 | 0 | 0 | 0 | 0 | 44 |
| *Kockovaella* | 0 | 0 | 0 | 3 | 0 | 0 | 0 |
| *Sporidiobolaceae* | 0 | 0 | 0 | 0 | 0 | 0 | 1 |
| *Malassezia restricta* | 0 | 0 | 0 | 0 | 0 | 0 | 2 |
| *Sporisorium graminicola* | 0 | 0 | 0 | 1 | 0 | 0 | 0 |
| *Mucorales* | 0 | 0 | 0 | 1 | 0 | 0 | 0 |
| *Salarias fasciatus* | 0 | 0 | 1 | 0 | 0 | 1 | 0 |
| *Oncorhynchus tshawytscha* | 0 | 0 | 0 | 7 | 0 | 0 | 0 |
| *Cyprinus carpio* | 0 | 0 | 4 | 0 | 8 | 51 | 4 |
| *Heterocephalus glaber* | 0 | 0 | 1 | 0 | 0 | 0 | 0 |
| *Ictidomys tridecemlineatus* | 1 | 4 | 19 | 0 | 1 | 28 | 1 |
| *Macaca fascicularis* | 0 | 0 | 0 | 0 | 0 | 0 | 1 |
| *Theropithecus gelada* | 0 | 0 | 0 | 0 | 0 | 0 | 1 |
| *Homo sapiens* | 1 | 3 | 0 | 5 | 3 | 1 | 650 |
| *Pan troglodytes* | 0 | 0 | 0 | 0 | 0 | 0 | 17 |
| *Pongo abelii* | 0 | 0 | 0 | 0 | 0 | 5 | 1 |
| *Vulpes vulpes* | 0 | 0 | 0 | 0 | 0 | 0 | 1 |
| *Lynx canadensis* | 0 | 0 | 0 | 0 | 0 | 0 | 1 |
| *Ovis canadensis* | 0 | 1 | 0 | 0 | 0 | 0 | 0 |
| *Odocoileus* | 0 | 2 | 0 | 0 | 0 | 0 | 0 |
| *Camelus ferus* | 0 | 0 | 0 | 0 | 0 | 2 | 0 |
| *Phyllostomidae* | 0 | 0 | 0 | 1 | 0 | 0 | 0 |
| *Aquila chrysaetos* | 0 | 0 | 0 | 8 | 0 | 0 | 0 |
| *Columba livia* | 0 | 0 | 0 | 3 | 0 | 0 | 0 |
| *Streptopelia turtur* | 0 | 0 | 0 | 29 | 0 | 0 | 0 |
| *Gallus gallus* | 0 | 0 | 1 | 0 | 0 | 0 | 0 |
| *Cyanistes caeruleus* | 0 | 0 | 0 | 1 | 0 | 0 | 0 |
| *Sturnus vulgaris* | 0 | 0 | 0 | 5 | 0 | 0 | 0 |
| *Geospiza fortis* | 0 | 0 | 0 | 1 | 0 | 0 | 0 |
| *Diphyllobothrium* | 0 | 3 | 0 | 0 | 0 | 84 | 0 |
| *Spirometra erinaceieuropaei* | 0 | 0 | 0 | 0 | 0 | 0 | 1 |
| *Brugia timori* | 0 | 0 | 0 | 1 | 0 | 0 | 0 |
| *Onchocerca ochengi* | 0 | 0 | 0 | 1 | 0 | 0 | 0 |
| *Nippostrongylus brasiliensis* | 0 | 0 | 0 | 0 | 1 | 0 | 0 |
| *Proasellus solanasi* | 0 | 0 | 0 | 0 | 0 | 1 | 0 |
| *Scaptodrosophila lebanonensis* | 0 | 0 | 0 | 0 | 0 | 1 | 0 |
| *Drosophila pseudoobscura* | 0 | 0 | 0 | 0 | 0 | 1 | 0 |
| *Sciaroidea* | 0 | 0 | 0 | 1 | 0 | 0 | 0 |
| *Culex pipiens* | 0 | 0 | 0 | 2 | 0 | 0 | 0 |
| *Camponotus floridanus* | 0 | 2 | 0 | 0 | 0 | 0 | 0 |
| *Diaspididae* | 0 | 0 | 0 | 1 | 0 | 0 | 0 |
| *Pontoscolex corethrurus* | 0 | 0 | 0 | 0 | 0 | 1 | 0 |
| *Cercomonadida* | 0 | 0 | 0 | 1 | 0 | 0 | 0 |
| *Stramenopiles* | 0 | 0 | 0 | 6 | 0 | 0 | 0 |
| *Volvox carteri* | 0 | 0 | 0 | 1 | 0 | 0 | 0 |
| *Micractinium conductrix* | 0 | 0 | 0 | 1 | 0 | 0 | 0 |
| *Coccomyxa subellipsoidea* | 0 | 0 | 0 | 1 | 0 | 0 | 0 |
| *Physcomitrella patens* | 0 | 0 | 0 | 0 | 0 | 1 | 0 |
| *Dioon* | 0 | 0 | 0 | 0 | 0 | 94 | 0 |
| *Pinus <subgenus>* | 0 | 0 | 0 | 1 | 0 | 0 | 0 |
| *Daucus carota* | 0 | 0 | 0 | 3 | 0 | 0 | 0 |
| *Lasthenia californica* | 0 | 0 | 0 | 0 | 0 | 3 | 0 |
| *Oreocharis mileensis* | 0 | 0 | 0 | 1 | 0 | 0 | 0 |
| *Fagales* | 0 | 0 | 0 | 14 | 0 | 0 | 0 |
| *Populus trichocarpa* | 0 | 0 | 0 | 2 | 0 | 0 | 0 |
| *Malus domestica* | 0 | 0 | 0 | 0 | 0 | 0 | 2 |
| *Oryza sativa* | 0 | 0 | 0 | 1 | 0 | 0 | 0 |
| *Triticum aestivum* | 0 | 0 | 0 | 0 | 0 | 0 | 1 |
| *Zea mays* | 0 | 0 | 0 | 2 | 0 | 0 | 0 |
| *Dioscorea rotundata* | 0 | 0 | 0 | 1 | 0 | 0 | 0 |
| **HABbaits1** | **HYBHAB_A21980_EBC** | **HYBHAB_A22301_EBC** | **HYBHAB_A22302_EBC** | **HYBHAB_A23351_EBC** | **HYBHAB_A23352_EBC** | **HYBHAB_A24029_EBC** | **HYBHAB_A24030_EBC** |
| *Stenamoeba* | 0 | 0 | 0 | 1 | 0 | 0 | 0 |
| *Opegrapha vulgata* | 0 | 0 | 0 | 0 | 0 | 0 | 1 |
| *Pleosporales* | 0 | 0 | 0 | 1 | 0 | 0 | 0 |
| *Eurotiales* | 0 | 0 | 0 | 1 | 0 | 0 | 0 |
| *Agaricomycetes incertae sedis* | 0 | 0 | 0 | 1 | 0 | 0 | 0 |
| *Malassezia restricta* | 0 | 0 | 0 | 0 | 0 | 0 | 1 |
| *Rhizophydiales* | 0 | 0 | 0 | 1 | 0 | 0 | 0 |
| *Cyprinus carpio* | 0 | 0 | 0 | 0 | 3 | 2 | 1 |
| *Ictidomys tridecemlineatus* | 0 | 0 | 3 | 0 | 0 | 0 | 0 |
| *Theropithecus gelada* | 0 | 0 | 0 | 0 | 0 | 0 | 1 |
| *Homo sapiens* | 0 | 0 | 0 | 1 | 0 | 0 | 83 |
| *Pongo abelii* | 0 | 0 | 0 | 0 | 0 | 1 | 0 |
| *Lynx canadensis* | 0 | 0 | 0 | 0 | 0 | 0 | 1 |
| *Aquila chrysaetos* | 0 | 0 | 0 | 1 | 0 | 0 | 0 |
| *Streptopelia turtur* | 0 | 0 | 0 | 1 | 0 | 0 | 0 |
| *Corvus brachyrhynchos* | 0 | 0 | 0 | 1 | 0 | 0 | 0 |
| *Zonotrichia albicollis* | 0 | 0 | 0 | 1 | 0 | 0 | 0 |
| *Diphyllobothrium* | 0 | 1 | 0 | 0 | 0 | 3 | 0 |
| *Rhabditidae* | 0 | 0 | 0 | 1 | 0 | 0 | 0 |
| *Harmonia axyridis* | 0 | 0 | 0 | 1 | 0 | 0 | 0 |
| *Sciaroidea* | 0 | 0 | 0 | 1 | 0 | 0 | 0 |
| *Diaspididae* | 0 | 0 | 0 | 1 | 0 | 0 | 0 |
| *Cercozoa* | 0 | 0 | 0 | 2 | 0 | 0 | 0 |
| *Stramenopiles* | 0 | 0 | 0 | 1 | 0 | 0 | 0 |
| *Chlorophyta* | 0 | 0 | 0 | 1 | 0 | 0 | 0 |
| *Dioon* | 0 | 0 | 0 | 0 | 0 | 6 | 0 |
| *Malus domestica* | 0 | 0 | 0 | 0 | 0 | 0 | 2 |
| *Triticum aestivum* | 0 | 0 | 0 | 0 | 0 | 0 | 1 |

**Supplementary Material Table 2.** Eukaryote taxa determined by HOPS (after running taxalist *(a)* ‘Eukaryota’) in extraction blank controls (EBCs) of Shotgun, Planktonbaits1 and HABbaits1. EBC taxa are listed separately for ancient and default output. EBC taxa were removed from downstream *sed*aDNA damage analyses (separately for each dataset).

| **Shotgun - Ancient** | **SG_21980A_EBC** | **SG_22301A_EBC** | **SG_22302A_EBC** | **SG_23351A_EBC** | **SG_23352A_EBC** | **SG_24029A_EBC** | **SG_24030A_EBC** |
| --- | --- | --- | --- | --- | --- | --- | --- |
| *Acrocephalus_arundinaceus* | 0 | 0 | 0 | 0 | 0 | 0 | 0 |
| *Acrogymnospermae* | 0 | 0 | 0 | 0 | 0 | 0 | 0 |
| *Acropora_digitifera* | 0 | 16 | 0 | 0 | 0 | 0 | 0 |
| *Agaricomycetes_incertae_sedis* | 0 | 0 | 0 | 0 | 0 | 0 | 0 |
| *Alternaria* | 0 | 0 | 0 | 0 | 0 | 0 | 0 |
| *Alternaria_alternata* | 0 | 0 | 0 | 0 | 0 | 0 | 0 |
| *Alternaria_arborescens* | 0 | 0 | 0 | 0 | 0 | 0 | 0 |
| *Alternaria_sect._Alternaria* | 0 | 0 | 0 | 0 | 0 | 0 | 0 |
| *Amniota* | 0 | 0 | 0 | 0 | 0 | 0 | 0 |
| *Aotus_nancymaae* | 0 | 0 | 0 | 0 | 0 | 0 | 0 |
| *Apteryx_australis* | 0 | 0 | 0 | 0 | 0 | 0 | 0 |
| *Aquila_chrysaetos* | 0 | 0 | 0 | 0 | 0 | 0 | 0 |
| *Archosauria* | 0 | 0 | 0 | 0 | 0 | 0 | 0 |
| *Ascomycota* | 0 | 0 | 0 | 0 | 0 | 0 | 0 |
| *Aureobasidium_pullulans* | 0 | 0 | 0 | 0 | 0 | 0 | 0 |
| *Aves* | 0 | 0 | 0 | 0 | 0 | 0 | 0 |
| *Beauveria_bassiana* | 0 | 0 | 0 | 0 | 0 | 0 | 0 |
| *Bilateria* | 0 | 0 | 1 | 4 | 1 | 0 | 0 |
| *Boreoeutheria* | 0 | 0 | 0 | 0 | 0 | 0 | 0 |
| *Bos* | 0 | 0 | 0 | 0 | 0 | 0 | 0 |
| *Bos_grunniens* | 0 | 0 | 0 | 0 | 0 | 0 | 0 |
| *Bos_indicus* | 0 | 0 | 0 | 0 | 0 | 0 | 0 |
| *Bos_indicus_x_Bos_taurus* | 0 | 0 | 0 | 0 | 0 | 0 | 0 |
| *Bos_mutus* | 0 | 0 | 0 | 0 | 0 | 0 | 0 |
| *Bos_taurus* | 0 | 0 | 0 | 0 | 0 | 0 | 0 |
| *Bovidae* | 0 | 0 | 0 | 0 | 0 | 0 | 0 |
| *Bovinae* | 0 | 0 | 0 | 0 | 0 | 0 | 0 |
| *Brugia_timori* | 0 | 0 | 0 | 0 | 0 | 0 | 0 |
| *Camelus_ferus* | 0 | 0 | 0 | 0 | 0 | 1 | 0 |
| *Camponotus_floridanus* | 0 | 0 | 0 | 0 | 0 | 0 | 0 |
| *Capra_hircus* | 0 | 0 | 0 | 0 | 0 | 0 | 0 |
| *Catarrhini* | 0 | 0 | 0 | 0 | 0 | 0 | 0 |
| *Cercomonadida* | 0 | 0 | 0 | 0 | 0 | 0 | 0 |
| *Cercozoa* | 0 | 0 | 0 | 0 | 0 | 0 | 0 |
| *Chaetomiaceae* | 0 | 0 | 0 | 0 | 0 | 0 | 0 |
| *Chaetomium_globosum* | 0 | 0 | 0 | 0 | 0 | 0 | 0 |
| *Chionanthus_rupicola* | 0 | 0 | 0 | 0 | 0 | 0 | 0 |
| *Chlamydomonadales* | 0 | 0 | 0 | 0 | 0 | 0 | 0 |
| *Chlorella_clade* | 0 | 0 | 0 | 0 | 0 | 0 | 0 |
| *Chlorococcum_tatrense* | 0 | 0 | 0 | 0 | 0 | 0 | 0 |
| *Citrus_sinensis* | 0 | 0 | 0 | 0 | 0 | 0 | 0 |
| *Clupea_harengus* | 0 | 0 | 0 | 0 | 0 | 0 | 0 |
| *Clupeocephala* | 0 | 0 | 0 | 0 | 0 | 1 | 0 |
| *Coccomyxa* | 0 | 0 | 0 | 0 | 0 | 0 | 0 |
| *Coccomyxa_sp._SUA001* | 0 | 0 | 0 | 0 | 0 | 0 | 0 |
| *Columba* | 0 | 0 | 0 | 0 | 0 | 0 | 0 |
| *Columba_livia* | 0 | 0 | 0 | 0 | 0 | 0 | 0 |
| *Columbidae* | 0 | 0 | 0 | 0 | 0 | 0 | 0 |
| *Conus_episcopatus* | 0 | 0 | 0 | 0 | 0 | 0 | 0 |
| *Cordyceps_militaris* | 0 | 0 | 0 | 0 | 0 | 0 | 0 |
| *Cordycipitaceae* | 0 | 0 | 0 | 0 | 0 | 0 | 0 |
| *Corvus_brachyrhynchos* | 0 | 0 | 0 | 0 | 0 | 0 | 0 |
| *Cryptococcus_amylolentus* | 0 | 0 | 0 | 0 | 0 | 0 | 0 |
| *Culex_pipiens* | 0 | 0 | 0 | 0 | 0 | 0 | 0 |
| *Cyanistes_caeruleus* | 0 | 0 | 0 | 1 | 0 | 0 | 0 |
| *Cyprinus_carpio* | 6 | 13 | 0 | 0 | 3 | 2 | 8 |
| *Daucus_carota* | 0 | 0 | 0 | 2 | 0 | 0 | 0 |
| *Debaryomyces_hansenii* | 0 | 0 | 0 | 0 | 0 | 0 | 0 |
| *Dendronephthya_gigantea* | 0 | 0 | 0 | 0 | 0 | 0 | 0 |
| *Deuterostomia* | 0 | 0 | 0 | 0 | 0 | 0 | 0 |
| *Dicrocoelium_dendriticum* | 0 | 5 | 0 | 56 | 0 | 0 | 0 |
| *Dikarya* | 0 | 0 | 0 | 0 | 0 | 0 | 0 |
| *Dioon* | 0 | 0 | 0 | 0 | 0 | 0 | 0 |
| *Diphyllobothrium* | 0 | 8 | 0 | 0 | 0 | 1 | 0 |
| *Drosophila_biarmipes* | 0 | 0 | 0 | 0 | 0 | 0 | 0 |
| *Drosophila_pseudoobscura* | 0 | 0 | 0 | 0 | 0 | 0 | 0 |
| *Eimeria_acervulina* | 0 | 0 | 0 | 0 | 0 | 0 | 0 |
| *Elaeis_guineensis* | 0 | 0 | 0 | 0 | 0 | 0 | 0 |
| *Embryophyta* | 0 | 0 | 0 | 0 | 0 | 0 | 0 |
| *Eukaryota* | 1 | 1 | 0 | 0 | 0 | 1 | 0 |
| *Eumetazoa* | 0 | 3 | 0 | 0 | 0 | 0 | 0 |
| *Euteleostomi* | 0 | 0 | 0 | 0 | 0 | 0 | 0 |
| *Eutheria* | 0 | 0 | 0 | 0 | 0 | 0 | 0 |
| *Fagaceae* | 0 | 0 | 0 | 0 | 0 | 0 | 0 |
| *Fagales* | 0 | 0 | 0 | 0 | 0 | 0 | 0 |
| *Falco_peregrinus* | 0 | 0 | 0 | 1 | 0 | 0 | 0 |
| *Filobasidium_uniguttulatum* | 0 | 0 | 0 | 0 | 0 | 0 | 0 |
| *Fungi_incertae_sedis* | 0 | 0 | 0 | 0 | 0 | 0 | 0 |
| *Fusarium* | 0 | 0 | 0 | 0 | 0 | 0 | 0 |
| *Fusarium_fujikuroi* | 0 | 0 | 0 | 0 | 0 | 0 | 0 |
| *Fusarium_graminearum* | 0 | 0 | 0 | 0 | 0 | 0 | 0 |
| *Fusarium_oxysporum* | 0 | 0 | 0 | 0 | 0 | 0 | 0 |
| *Fusarium_pseudograminearum* | 0 | 0 | 0 | 0 | 0 | 0 | 0 |
| *Fusarium_sambucinum_species_complex* | 0 | 0 | 0 | 0 | 0 | 0 | 0 |
| *Fusarium_verticillioides* | 0 | 0 | 0 | 0 | 0 | 0 | 0 |
| *Gallus_gallus* | 0 | 0 | 0 | 0 | 0 | 0 | 0 |
| *Harmonia_axyridis* | 0 | 0 | 0 | 0 | 0 | 0 | 0 |
| *Heterocephalus_glaber* | 0 | 0 | 0 | 0 | 1 | 0 | 0 |
| *Hominidae* | 0 | 0 | 0 | 1 | 0 | 0 | 0 |
| *Homininae* | 0 | 1 | 0 | 0 | 0 | 0 | 0 |
| *Hominoidea* | 0 | 0 | 0 | 0 | 0 | 0 | 0 |
| *Homo_sapiens* | 0 | 0 | 0 | 0 | 0 | 0 | 0 |
| *Hyalella_azteca* | 0 | 0 | 0 | 0 | 0 | 0 | 0 |
| *Hypocreales* | 0 | 0 | 0 | 0 | 0 | 0 | 0 |
| *Ictidomys_tridecemlineatus* | 10 | 2 | 0 | 0 | 3 | 0 | 1 |
| *Kockovaella* | 0 | 0 | 0 | 0 | 0 | 0 | 0 |
| *Larimichthys_crocea* | 0 | 0 | 0 | 0 | 0 | 0 | 0 |
| *Lasthenia_californica* | 1 | 0 | 0 | 0 | 0 | 0 | 0 |
| *Lecanicillium* | 0 | 0 | 0 | 0 | 1 | 0 | 0 |
| *Macaca_fascicularis* | 0 | 0 | 0 | 0 | 0 | 0 | 0 |
| *Malpighiales* | 0 | 0 | 0 | 0 | 0 | 0 | 0 |
| *Mesangiospermae* | 0 | 0 | 0 | 0 | 0 | 0 | 0 |
| *Micractinium_conductrix* | 0 | 0 | 0 | 0 | 0 | 0 | 0 |
| *Moina_brachiata* | 0 | 0 | 0 | 0 | 0 | 0 | 0 |
| *Monoraphidium_neglectum* | 0 | 0 | 0 | 0 | 0 | 0 | 0 |
| *Mychonastes* | 0 | 0 | 0 | 0 | 0 | 0 | 0 |
| *Mychonastes_homosphaera* | 0 | 0 | 0 | 0 | 0 | 0 | 0 |
| *Neognathae* | 0 | 0 | 0 | 1 | 0 | 0 | 0 |
| *Nepenthes_ventricosa_x_Nepenthes_alata* | 0 | 0 | 0 | 0 | 0 | 0 | 0 |
| *Nippostrongylus_brasiliensis* | 0 | 0 | 0 | 0 | 0 | 0 | 0 |
| *Odocoileus* | 0 | 1 | 0 | 0 | 0 | 0 | 0 |
| *Odocoileus_hemionus* | 0 | 0 | 0 | 0 | 0 | 0 | 0 |
| *Odocoileus_virginianus* | 0 | 0 | 0 | 0 | 0 | 0 | 0 |
| *Oncorhynchus_nerka* | 0 | 0 | 0 | 0 | 0 | 0 | 0 |
| *Oncorhynchus_tshawytscha* | 0 | 0 | 0 | 2 | 0 | 0 | 0 |
| *Opisthokonta* | 0 | 0 | 0 | 0 | 0 | 0 | 0 |
| *Oryza_sativa* | 0 | 0 | 0 | 0 | 0 | 0 | 0 |
| *Ostrinia_furnacalis* | 0 | 0 | 0 | 1 | 0 | 0 | 0 |
| *Otomorpha* | 0 | 0 | 0 | 0 | 0 | 0 | 0 |
| *Ovis_canadensis* | 0 | 0 | 0 | 0 | 0 | 0 | 0 |
| *Pan_troglodytes* | 0 | 0 | 0 | 0 | 0 | 0 | 0 |
| *Panthera_pardus* | 0 | 0 | 0 | 0 | 0 | 0 | 0 |
| *Parasteatoda_tepidariorum* | 0 | 0 | 0 | 0 | 0 | 0 | 0 |
| *Parus_major* | 0 | 0 | 0 | 0 | 0 | 0 | 0 |
| *Passeriformes* | 0 | 0 | 0 | 0 | 0 | 0 | 0 |
| *Pecora* | 1 | 0 | 0 | 0 | 0 | 0 | 0 |
| *Penicillium* | 0 | 0 | 0 | 0 | 0 | 0 | 0 |
| *Penicillium_chrysogenum* | 0 | 0 | 0 | 0 | 0 | 0 | 0 |
| *Penicillium_expansum* | 0 | 0 | 0 | 0 | 0 | 0 | 0 |
| *Pentapetalae* | 0 | 0 | 0 | 0 | 0 | 0 | 0 |
| *Phaeoacremonium_minimum* | 0 | 0 | 0 | 0 | 0 | 0 | 0 |
| *Phaseolus* | 0 | 0 | 0 | 0 | 0 | 0 | 0 |
| *Physcomitrella_patens* | 0 | 0 | 0 | 0 | 0 | 0 | 0 |
| *Physochlaina_orientalis* | 0 | 0 | 0 | 0 | 0 | 0 | 0 |
| *Pinus* | 0 | 0 | 0 | 0 | 0 | 0 | 0 |
| *Pinus* | 0 | 0 | 0 | 0 | 0 | 0 | 0 |
| *Pinus_taeda* | 0 | 0 | 0 | 0 | 0 | 0 | 0 |
| *Plasmodium_berghei* | 0 | 0 | 0 | 0 | 0 | 0 | 0 |
| *Plasmodium_vivax* | 0 | 0 | 0 | 0 | 0 | 0 | 0 |
| *Pleosporales* | 0 | 0 | 0 | 0 | 0 | 0 | 0 |
| *Poales* | 0 | 0 | 0 | 0 | 0 | 0 | 0 |
| *Pongo_abelii* | 0 | 2 | 0 | 0 | 0 | 0 | 0 |
| *Populus* | 0 | 0 | 0 | 0 | 0 | 0 | 0 |
| *Populus_trichocarpa* | 0 | 0 | 0 | 0 | 0 | 0 | 0 |
| *Primates* | 0 | 0 | 0 | 0 | 0 | 0 | 0 |
| *Proasellus_solanasi* | 0 | 0 | 0 | 0 | 0 | 0 | 0 |
| *Psylloidea* | 0 | 0 | 0 | 0 | 0 | 0 | 0 |
| *Pyrus_x_bretschneideri* | 0 | 0 | 0 | 0 | 0 | 0 | 0 |
| *Quercus_suber* | 0 | 0 | 0 | 0 | 0 | 0 | 0 |
| *Rhodamnia_argentea* | 0 | 0 | 0 | 0 | 0 | 0 | 0 |
| *Rhopalosiphum_maidis* | 0 | 0 | 0 | 0 | 0 | 0 | 0 |
| *Saccharomyces_cerevisiae* | 0 | 0 | 0 | 0 | 0 | 0 | 0 |
| *Saccharomycetales* | 0 | 0 | 0 | 0 | 0 | 0 | 0 |
| *Salarias_fasciatus* | 0 | 0 | 0 | 0 | 0 | 0 | 0 |
| *Saprolegnia_parasitica* | 0 | 0 | 0 | 0 | 0 | 0 | 0 |
| *Serinus_canaria* | 0 | 0 | 0 | 0 | 0 | 0 | 0 |
| *Simiiformes* | 0 | 0 | 0 | 0 | 0 | 0 | 0 |
| *Sinocyclocheilus_anshuiensis* | 0 | 0 | 0 | 0 | 0 | 0 | 0 |
| *Sordariales* | 0 | 0 | 0 | 0 | 0 | 0 | 0 |
| *Sordariomycetidae* | 0 | 0 | 0 | 0 | 0 | 0 | 0 |
| *Spermatophyta* | 0 | 0 | 0 | 0 | 0 | 0 | 0 |
| *Sporidiobolaceae* | 0 | 0 | 0 | 0 | 0 | 0 | 0 |
| *Sporisorium_graminicola* | 0 | 0 | 0 | 0 | 0 | 0 | 0 |
| *Stenamoeba* | 0 | 0 | 0 | 0 | 0 | 0 | 0 |
| *Stereum_hirsutum* | 0 | 0 | 0 | 0 | 0 | 0 | 0 |
| *Streptopelia_turtur* | 0 | 0 | 0 | 4 | 0 | 0 | 0 |
| *Strongyloides_venezuelensis* | 0 | 0 | 0 | 0 | 0 | 0 | 0 |
| *Sturnus* | 0 | 0 | 0 | 0 | 0 | 0 | 0 |
| *Sturnus_vulgaris* | 0 | 0 | 0 | 0 | 0 | 0 | 0 |
| *Taeniopygia_guttata* | 0 | 0 | 0 | 0 | 0 | 0 | 0 |
| *Tetrapoda* | 0 | 0 | 0 | 0 | 0 | 0 | 0 |
| *Thelebolaceae* | 0 | 0 | 0 | 0 | 0 | 0 | 0 |
| *Theobroma_cacao* | 0 | 1 | 0 | 0 | 0 | 0 | 0 |
| *Trametes_versicolor* | 0 | 0 | 0 | 0 | 0 | 0 | 0 |
| *Trebouxiophyceae* | 0 | 0 | 0 | 0 | 0 | 0 | 0 |
| *Tremellomycetes* | 0 | 0 | 0 | 0 | 0 | 0 | 0 |
| *Trichobilharzia_regenti* | 0 | 0 | 0 | 0 | 0 | 0 | 0 |
| *Trichoderma* | 0 | 0 | 0 | 0 | 0 | 0 | 0 |
| *Trichosporon_asahii* | 0 | 0 | 0 | 0 | 0 | 0 | 0 |
| *Triticinae* | 0 | 0 | 0 | 0 | 0 | 0 | 0 |
| *Triticum* | 0 | 0 | 0 | 0 | 0 | 0 | 0 |
| *Triticum_aestivum* | 0 | 0 | 0 | 0 | 0 | 0 | 0 |
| *Triticum_monococcum* | 0 | 0 | 0 | 0 | 0 | 0 | 0 |
| *Venturia_effusa* | 0 | 0 | 0 | 0 | 0 | 0 | 0 |
| *Xylobolus_sp._101_OA-2013* | 0 | 0 | 0 | 0 | 0 | 0 | 0 |
| *Zea_mays* | 0 | 0 | 0 | 0 | 0 | 0 | 0 |
| *Zonotrichia_albicollis* | 0 | 0 | 0 | 0 | 0 | 0 | 0 |
| *fabids* | 0 | 0 | 0 | 0 | 0 | 0 | 0 |
| *leotiomyceta* | 0 | 0 | 0 | 0 | 0 | 0 | 0 |
| **Shotgun - Default** | **SG_21980A_EBC** | **SG_22301A_EBC** | **SG_22302A_EBC** | **SG_23351A_EBC** | **SG_23352A_EBC** | **SG_24029A_EBC** | **SG_24030A_EBC** |
| *Acrocephalus_arundinaceus* | 0 | 0 | 0 | 1 | 0 | 0 | 0 |
| *Acrogymnospermae* | 0 | 0 | 0 | 1 | 0 | 0 | 0 |
| *Acropora_digitifera* | 0 | 25 | 5 | 0 | 0 | 0 | 0 |
| *Agaricomycetes_incertae_sedis* | 0 | 0 | 0 | 1 | 0 | 0 | 0 |
| *Alternaria* | 0 | 0 | 0 | 1 | 0 | 0 | 0 |
| *Alternaria_alternata* | 0 | 0 | 0 | 2 | 0 | 0 | 0 |
| *Alternaria_arborescens* | 0 | 0 | 0 | 1 | 0 | 0 | 0 |
| *Alternaria_sect._Alternaria* | 0 | 0 | 0 | 1 | 0 | 0 | 0 |
| *Amniota* | 0 | 0 | 0 | 2 | 0 | 0 | 0 |
| *Aotus_nancymaae* | 0 | 0 | 0 | 0 | 0 | 0 | 1 |
| *Apteryx_australis* | 0 | 4 | 0 | 1 | 0 | 0 | 0 |
| *Aquila_chrysaetos* | 0 | 0 | 0 | 33 | 0 | 0 | 0 |
| *Archosauria* | 0 | 0 | 0 | 1 | 0 | 0 | 0 |
| *Ascomycota* | 0 | 0 | 0 | 3 | 0 | 0 | 0 |
| *Aureobasidium_pullulans* | 0 | 0 | 0 | 1 | 0 | 0 | 0 |
| *Aves* | 0 | 0 | 0 | 8 | 0 | 0 | 0 |
| *Beauveria_bassiana* | 0 | 0 | 0 | 0 | 1 | 0 | 0 |
| *Bilateria* | 5 | 21 | 3 | 8 | 11 | 7 | 22 |
| *Boreoeutheria* | 0 | 0 | 0 | 4 | 0 | 0 | 1 |
| *Bos* | 1 | 0 | 0 | 0 | 0 | 0 | 0 |
| *Bos_grunniens* | 0 | 0 | 0 | 0 | 0 | 0 | 0 |
| *Bos_indicus* | 0 | 0 | 0 | 0 | 0 | 0 | 0 |
| *Bos_indicus_x_Bos_taurus* | 0 | 0 | 0 | 0 | 0 | 0 | 0 |
| *Bos_mutus* | 0 | 4 | 0 | 0 | 1 | 0 | 0 |
| *Bos_taurus* | 3 | 0 | 0 | 0 | 0 | 0 | 0 |
| *Bovidae* | 0 | 6 | 0 | 0 | 1 | 0 | 0 |
| *Bovinae* | 0 | 0 | 0 | 0 | 0 | 0 | 0 |
| *Brugia_timori* | 0 | 0 | 0 | 6 | 0 | 0 | 0 |
| *Camelus_ferus* | 13 | 7 | 0 | 1 | 0 | 8 | 0 |
| *Camponotus_floridanus* | 0 | 8 | 0 | 0 | 0 | 0 | 0 |
| *Capra_hircus* | 0 | 0 | 0 | 1 | 0 | 0 | 0 |
| *Catarrhini* | 0 | 0 | 0 | 0 | 1 | 0 | 4 |
| *Cercomonadida* | 0 | 0 | 0 | 1 | 0 | 0 | 0 |
| *Cercozoa* | 0 | 0 | 0 | 1 | 0 | 0 | 0 |
| *Chaetomiaceae* | 0 | 0 | 0 | 1 | 0 | 0 | 0 |
| *Chaetomium_globosum* | 0 | 0 | 0 | 1 | 0 | 0 | 0 |
| *Chionanthus_rupicola* | 0 | 0 | 0 | 1 | 0 | 0 | 0 |
| *Chlamydomonadales* | 0 | 0 | 0 | 3 | 0 | 0 | 0 |
| *Chlorella_clade* | 0 | 0 | 0 | 1 | 0 | 0 | 0 |
| *Chlorococcum_tatrense* | 0 | 0 | 0 | 4 | 0 | 0 | 0 |
| *Citrus_sinensis* | 0 | 0 | 0 | 1 | 0 | 0 | 0 |
| *Clupea_harengus* | 0 | 2 | 0 | 0 | 0 | 0 | 0 |
| *Clupeocephala* | 0 | 0 | 0 | 0 | 0 | 4 | 0 |
| *Coccomyxa* | 0 | 0 | 0 | 1 | 0 | 0 | 0 |
| *Coccomyxa_sp._SUA001* | 0 | 0 | 0 | 1 | 0 | 0 | 0 |
| *Columba* | 0 | 0 | 0 | 2 | 0 | 0 | 0 |
| *Columba_livia* | 0 | 0 | 0 | 19 | 0 | 0 | 0 |
| *Columbidae* | 0 | 0 | 0 | 1 | 0 | 0 | 0 |
| *Conus_episcopatus* | 0 | 0 | 0 | 1 | 0 | 0 | 0 |
| *Cordyceps_militaris* | 0 | 1 | 0 | 0 | 3 | 0 | 0 |
| *Cordycipitaceae* | 0 | 1 | 0 | 0 | 2 | 0 | 0 |
| *Corvus_brachyrhynchos* | 0 | 0 | 0 | 3 | 0 | 0 | 0 |
| *Cryptococcus_amylolentus* | 0 | 0 | 0 | 1 | 0 | 0 | 0 |
| *Culex_pipiens* | 0 | 0 | 0 | 1 | 0 | 0 | 0 |
| *Cyanistes_caeruleus* | 0 | 0 | 0 | 2 | 0 | 0 | 0 |
| *Cyprinus_carpio* | 24 | 569 | 10 | 41 | 127 | 123 | 27 |
| *Daucus_carota* | 0 | 0 | 0 | 12 | 0 | 0 | 0 |
| *Debaryomyces_hansenii* | 0 | 1 | 0 | 0 | 1 | 0 | 0 |
| *Dendronephthya_gigantea* | 0 | 0 | 0 | 1 | 0 | 0 | 0 |
| *Deuterostomia* | 0 | 0 | 0 | 1 | 0 | 0 | 0 |
| *Dicrocoelium_dendriticum* | 0 | 8 | 0 | 58 | 0 | 2 | 0 |
| *Dikarya* | 0 | 2 | 0 | 0 | 0 | 0 | 0 |
| *Dioon* | 8 | 33 | 0 | 3 | 0 | 74 | 0 |
| *Diphyllobothrium* | 0 | 322 | 0 | 1 | 1 | 30 | 0 |
| *Drosophila_biarmipes* | 0 | 0 | 0 | 1 | 0 | 0 | 0 |
| *Drosophila_pseudoobscura* | 0 | 2 | 0 | 0 | 0 | 3 | 0 |
| *Eimeria_acervulina* | 0 | 1 | 1 | 2 | 0 | 0 | 0 |
| *Elaeis_guineensis* | 0 | 0 | 0 | 4 | 0 | 0 | 0 |
| *Embryophyta* | 2 | 7 | 0 | 0 | 0 | 13 | 0 |
| *Eukaryota* | 10 | 66 | 3 | 1 | 1 | 78 | 1 |
| *Eumetazoa* | 0 | 31 | 12 | 0 | 0 | 0 | 0 |
| *Euteleostomi* | 4 | 15 | 0 | 2 | 1 | 18 | 1 |
| *Eutheria* | 0 | 0 | 0 | 0 | 0 | 1 | 0 |
| *Fagaceae* | 0 | 0 | 0 | 1 | 0 | 0 | 0 |
| *Fagales* | 0 | 0 | 0 | 1 | 0 | 0 | 0 |
| *Falco_peregrinus* | 0 | 0 | 0 | 1 | 0 | 0 | 0 |
| *Filobasidium_uniguttulatum* | 0 | 0 | 0 | 1 | 0 | 0 | 0 |
| *Fungi_incertae_sedis* | 0 | 0 | 0 | 1 | 0 | 0 | 0 |
| *Fusarium* | 1 | 0 | 0 | 1 | 0 | 0 | 0 |
| *Fusarium_fujikuroi* | 0 | 0 | 0 | 1 | 0 | 0 | 0 |
| *Fusarium_graminearum* | 0 | 0 | 0 | 2 | 0 | 0 | 0 |
| *Fusarium_oxysporum* | 1 | 0 | 0 | 0 | 0 | 0 | 0 |
| *Fusarium_pseudograminearum* | 0 | 0 | 0 | 1 | 0 | 0 | 0 |
| *Fusarium_sambucinum_species_complex* | 0 | 0 | 0 | 1 | 0 | 0 | 0 |
| *Fusarium_verticillioides* | 0 | 0 | 0 | 7 | 0 | 0 | 0 |
| *Gallus_gallus* | 0 | 0 | 0 | 1 | 0 | 0 | 0 |
| *Harmonia_axyridis* | 0 | 0 | 0 | 2 | 0 | 0 | 0 |
| *Heterocephalus_glaber* | 22 | 4 | 3 | 5 | 1 | 1 | 0 |
| *Hominidae* | 0 | 0 | 0 | 1 | 0 | 0 | 6 |
| *Homininae* | 6 | 10 | 0 | 5 | 2 | 0 | 31 |
| *Hominoidea* | 0 | 0 | 0 | 0 | 0 | 0 | 1 |
| *Homo_sapiens* | 9 | 17 | 0 | 9 | 21 | 0 | 126 |
| *Hyalella_azteca* | 0 | 0 | 0 | 1 | 0 | 0 | 0 |
| *Hypocreales* | 0 | 0 | 0 | 0 | 0 | 0 | 1 |
| *Ictidomys_tridecemlineatus* | 153 | 82 | 8 | 1 | 111 | 15 | 1 |
| *Kockovaella* | 0 | 0 | 0 | 1 | 0 | 0 | 0 |
| *Larimichthys_crocea* | 1 | 5 | 0 | 0 | 1 | 0 | 0 |
| *Lasthenia_californica* | 22 | 0 | 0 | 0 | 0 | 0 | 0 |
| *Lecanicillium* | 0 | 0 | 0 | 0 | 3 | 0 | 0 |
| *Macaca_fascicularis* | 0 | 1 | 0 | 0 | 0 | 0 | 0 |
| *Malpighiales* | 0 | 0 | 0 | 1 | 0 | 0 | 0 |
| *Mesangiospermae* | 0 | 0 | 0 | 1 | 0 | 0 | 0 |
| *Micractinium_conductrix* | 0 | 0 | 0 | 1 | 0 | 0 | 0 |
| *Moina_brachiata* | 0 | 0 | 0 | 0 | 0 | 1 | 0 |
| *Monoraphidium_neglectum* | 0 | 0 | 0 | 1 | 0 | 0 | 0 |
| *Mychonastes* | 0 | 0 | 0 | 1 | 0 | 0 | 0 |
| *Mychonastes_homosphaera* | 0 | 0 | 0 | 2 | 0 | 0 | 0 |
| *Neognathae* | 0 | 0 | 0 | 27 | 0 | 0 | 0 |
| *Nepenthes_ventricosa_x_Nepenthes_alata* | 0 | 0 | 0 | 1 | 0 | 0 | 0 |
| *Nippostrongylus_brasiliensis* | 0 | 2 | 0 | 0 | 39 | 4 | 0 |
| *Odocoileus* | 13 | 1 | 0 | 0 | 0 | 0 | 0 |
| *Odocoileus_hemionus* | 0 | 1 | 0 | 0 | 0 | 0 | 0 |
| *Odocoileus_virginianus* | 0 | 1 | 0 | 0 | 0 | 0 | 0 |
| *Oncorhynchus_nerka* | 0 | 0 | 0 | 0 | 1 | 0 | 0 |
| *Oncorhynchus_tshawytscha* | 0 | 0 | 0 | 25 | 0 | 0 | 0 |
| *Opisthokonta* | 0 | 0 | 0 | 1 | 0 | 0 | 1 |
| *Oryza_sativa* | 0 | 0 | 0 | 1 | 0 | 0 | 0 |
| *Ostrinia_furnacalis* | 0 | 0 | 0 | 1 | 0 | 0 | 0 |
| *Otomorpha* | 0 | 2 | 0 | 1 | 0 | 1 | 0 |
| *Ovis_canadensis* | 0 | 1 | 0 | 0 | 0 | 0 | 0 |
| *Pan_troglodytes* | 0 | 0 | 0 | 1 | 0 | 0 | 3 |
| *Panthera_pardus* | 0 | 1 | 0 | 0 | 0 | 1 | 0 |
| *Parasteatoda_tepidariorum* | 0 | 1 | 0 | 0 | 0 | 0 | 0 |
| *Parus_major* | 0 | 0 | 0 | 1 | 0 | 0 | 0 |
| *Passeriformes* | 0 | 0 | 0 | 33 | 0 | 0 | 0 |
| *Pecora* | 2 | 0 | 0 | 0 | 0 | 0 | 0 |
| *Penicillium* | 0 | 0 | 0 | 1 | 0 | 0 | 0 |
| *Penicillium_chrysogenum* | 0 | 0 | 0 | 1 | 0 | 0 | 0 |
| *Penicillium_expansum* | 0 | 0 | 0 | 1 | 0 | 0 | 0 |
| *Pentapetalae* | 0 | 0 | 0 | 7 | 0 | 0 | 0 |
| *Phaeoacremonium_minimum* | 0 | 0 | 0 | 2 | 0 | 0 | 0 |
| *Phaseolus* | 0 | 1 | 0 | 0 | 0 | 0 | 0 |
| *Physcomitrella_patens* | 1 | 0 | 0 | 0 | 0 | 0 | 0 |
| *Physochlaina_orientalis* | 0 | 0 | 0 | 1 | 0 | 0 | 0 |
| *Pinus* | 0 | 0 | 0 | 2 | 0 | 0 | 0 |
| *Pinus* | 0 | 0 | 0 | 0 | 0 | 0 | 0 |
| *Pinus_taeda* | 0 | 0 | 0 | 8 | 0 | 0 | 0 |
| *Plasmodium_berghei* | 0 | 0 | 0 | 0 | 0 | 1 | 0 |
| *Plasmodium_vivax* | 0 | 0 | 0 | 0 | 2 | 0 | 0 |
| *Pleosporales* | 0 | 0 | 0 | 1 | 0 | 0 | 0 |
| *Poales* | 0 | 1 | 0 | 0 | 0 | 0 | 0 |
| *Pongo_abelii* | 0 | 12 | 0 | 0 | 0 | 9 | 0 |
| *Populus* | 0 | 0 | 0 | 1 | 0 | 0 | 0 |
| *Populus_trichocarpa* | 0 | 0 | 0 | 3 | 0 | 0 | 0 |
| *Primates* | 0 | 0 | 0 | 1 | 0 | 0 | 0 |
| *Proasellus_solanasi* | 0 | 3 | 0 | 1 | 0 | 4 | 0 |
| *Psylloidea* | 0 | 0 | 0 | 1 | 0 | 0 | 0 |
| *Pyrus_x_bretschneideri* | 0 | 0 | 0 | 0 | 0 | 0 | 1 |
| *Quercus_suber* | 0 | 0 | 0 | 2 | 0 | 0 | 0 |
| *Rhodamnia_argentea* | 0 | 1 | 0 | 0 | 0 | 0 | 0 |
| *Rhopalosiphum_maidis* | 0 | 0 | 0 | 2 | 0 | 0 | 0 |
| *Saccharomyces_cerevisiae* | 0 | 1 | 0 | 0 | 0 | 0 | 0 |
| *Saccharomycetales* | 0 | 0 | 0 | 0 | 0 | 0 | 1 |
| *Salarias_fasciatus* | 0 | 1 | 0 | 0 | 0 | 2 | 0 |
| *Saprolegnia_parasitica* | 0 | 0 | 0 | 1 | 0 | 0 | 0 |
| *Serinus_canaria* | 0 | 0 | 0 | 2 | 0 | 0 | 0 |
| *Simiiformes* | 1 | 0 | 0 | 1 | 1 | 0 | 1 |
| *Sinocyclocheilus_anshuiensis* | 0 | 1 | 0 | 0 | 0 | 0 | 0 |
| *Sordariales* | 0 | 0 | 0 | 1 | 0 | 0 | 0 |
| *Sordariomycetidae* | 0 | 0 | 0 | 1 | 0 | 0 | 0 |
| *Spermatophyta* | 0 | 1 | 0 | 0 | 0 | 0 | 0 |
| *Sporidiobolaceae* | 0 | 0 | 0 | 0 | 0 | 0 | 1 |
| *Sporisorium_graminicola* | 0 | 0 | 0 | 1 | 0 | 0 | 0 |
| *Stenamoeba* | 0 | 0 | 0 | 1 | 0 | 0 | 0 |
| *Stereum_hirsutum* | 0 | 0 | 0 | 12 | 0 | 0 | 0 |
| *Streptopelia_turtur* | 0 | 0 | 0 | 115 | 0 | 0 | 0 |
| *Strongyloides_venezuelensis* | 0 | 0 | 0 | 1 | 0 | 0 | 0 |
| *Sturnus* | 0 | 0 | 0 | 1 | 0 | 0 | 0 |
| *Sturnus_vulgaris* | 0 | 0 | 0 | 34 | 0 | 0 | 0 |
| *Taeniopygia_guttata* | 0 | 0 | 0 | 6 | 0 | 0 | 0 |
| *Tetrapoda* | 0 | 0 | 0 | 1 | 0 | 0 | 0 |
| *Thelebolaceae* | 0 | 0 | 0 | 1 | 0 | 0 | 0 |
| *Theobroma_cacao* | 0 | 5 | 0 | 0 | 0 | 0 | 0 |
| *Trametes_versicolor* | 0 | 0 | 0 | 2 | 0 | 0 | 0 |
| *Trebouxiophyceae* | 0 | 0 | 0 | 1 | 0 | 0 | 0 |
| *Tremellomycetes* | 0 | 0 | 0 | 1 | 0 | 0 | 0 |
| *Trichobilharzia_regenti* | 0 | 0 | 0 | 0 | 1 | 0 | 0 |
| *Trichoderma* | 0 | 2 | 0 | 0 | 0 | 0 | 0 |
| *Trichosporon_asahii* | 1 | 0 | 0 | 1 | 0 | 0 | 0 |
| *Triticinae* | 0 | 2 | 0 | 1 | 1 | 0 | 0 |
| *Triticum* | 0 | 1 | 0 | 0 | 0 | 0 | 0 |
| *Triticum_aestivum* | 0 | 1 | 0 | 0 | 0 | 0 | 0 |
| *Triticum_monococcum* | 0 | 0 | 0 | 0 | 0 | 0 | 1 |
| *Venturia_effusa* | 0 | 0 | 0 | 1 | 0 | 0 | 0 |
| *Xylobolus_sp._101_OA-2013* | 0 | 0 | 0 | 2 | 0 | 0 | 0 |
| *Zea_mays* | 0 | 0 | 0 | 3 | 0 | 0 | 0 |
| *Zonotrichia_albicollis* | 0 | 0 | 0 | 1 | 0 | 0 | 0 |
| *fabids* | 0 | 0 | 0 | 2 | 0 | 0 | 0 |
| *leotiomyceta* | 0 | 0 | 0 | 2 | 0 | 0 | 0 |
| **Planktonbaits1 - Ancient** | **HYB18SV9_A21980_EBC** | **HYB18SV9_A22301_EBC** | **HYB18SV9_A22302_EBC** | **HYB18SV9_A23351_EBC** | **HYB18SV9_A23352_EBC** | **HYB18SV9_A24029_EBC** | **HYB18SV9_A24030_EBC** |
| *Acrogymnospermae* | 0 | 0 | 0 | 0 | 0 | 0 | 0 |
| *Agaricomycetes_incertae_sedis* | 0 | 0 | 0 | 0 | 0 | 0 | 0 |
| *Agaricomycotina* | 0 | 0 | 0 | 0 | 0 | 0 | 0 |
| *Alternaria_solani* | 0 | 0 | 0 | 0 | 0 | 0 | 0 |
| *Amniota* | 0 | 0 | 0 | 0 | 0 | 0 | 0 |
| *Amygdaloideae* | 0 | 0 | 0 | 0 | 0 | 0 | 0 |
| *Aquila_chrysaetos* | 0 | 0 | 0 | 0 | 0 | 0 | 0 |
| *Ascomycota* | 0 | 0 | 0 | 0 | 0 | 0 | 0 |
| *Aureobasidium_pullulans* | 0 | 0 | 0 | 0 | 0 | 0 | 0 |
| *Aves* | 0 | 0 | 0 | 0 | 0 | 0 | 0 |
| *Basidiomycota* | 0 | 0 | 0 | 0 | 0 | 0 | 0 |
| *Bilateria* | 0 | 0 | 0 | 0 | 0 | 1 | 5 |
| *Boreoeutheria* | 0 | 0 | 0 | 0 | 0 | 0 | 0 |
| *Bos* | 0 | 0 | 0 | 0 | 0 | 0 | 0 |
| *Bos_indicus* | 0 | 0 | 0 | 0 | 0 | 0 | 0 |
| *Bos_indicus_x_Bos_taurus* | 0 | 0 | 0 | 0 | 0 | 0 | 0 |
| *Bos_mutus* | 0 | 0 | 0 | 0 | 0 | 0 | 0 |
| *Bos_taurus* | 0 | 0 | 0 | 0 | 0 | 0 | 0 |
| *Bovidae* | 0 | 0 | 0 | 0 | 0 | 0 | 0 |
| *Bovinae* | 0 | 0 | 0 | 0 | 0 | 0 | 0 |
| *Brugia_timori* | 0 | 0 | 0 | 0 | 0 | 0 | 0 |
| *Camelus_ferus* | 0 | 0 | 0 | 0 | 0 | 0 | 0 |
| *Camponotus_floridanus* | 0 | 0 | 0 | 0 | 0 | 0 | 0 |
| *Catarrhini* | 0 | 0 | 0 | 0 | 0 | 0 | 3 |
| *Cercomonadida* | 0 | 0 | 0 | 0 | 0 | 0 | 0 |
| *Cercopithecinae* | 0 | 0 | 0 | 0 | 0 | 0 | 0 |
| *Cercozoa* | 0 | 0 | 0 | 0 | 0 | 0 | 0 |
| *Chlamydomonadales* | 0 | 0 | 0 | 0 | 0 | 0 | 0 |
| *Coccomyxa* | 0 | 0 | 0 | 0 | 0 | 0 | 0 |
| *Coccomyxa_subellipsoidea* | 0 | 0 | 0 | 0 | 0 | 0 | 0 |
| *Columba* | 0 | 0 | 0 | 0 | 0 | 0 | 0 |
| *Columba_livia* | 0 | 0 | 0 | 0 | 0 | 0 | 0 |
| *Columbidae* | 0 | 0 | 0 | 0 | 0 | 0 | 0 |
| *Cryptococcus_neoformans* | 0 | 0 | 0 | 0 | 0 | 0 | 0 |
| *Culex_pipiens* | 0 | 0 | 0 | 0 | 0 | 0 | 0 |
| *Cyanistes_caeruleus* | 0 | 0 | 0 | 0 | 0 | 0 | 0 |
| *Cyprinus_carpio* | 0 | 0 | 0 | 0 | 0 | 3 | 2 |
| *Daucus_carota* | 0 | 0 | 0 | 0 | 0 | 0 | 0 |
| *Deuterostomia* | 0 | 0 | 0 | 0 | 0 | 0 | 0 |
| *Diaspididae* | 0 | 0 | 0 | 0 | 0 | 0 | 0 |
| *Dioon* | 0 | 0 | 0 | 0 | 0 | 1 | 0 |
| *Dioscorea_rotundata* | 0 | 0 | 0 | 0 | 0 | 0 | 0 |
| *Diphyllobothrium* | 0 | 0 | 0 | 0 | 0 | 0 | 0 |
| *Dothideomycetes* | 0 | 0 | 0 | 0 | 0 | 0 | 0 |
| *Drosophila_pseudoobscura* | 0 | 0 | 0 | 0 | 0 | 0 | 0 |
| *Embryophyta* | 0 | 0 | 0 | 0 | 0 | 0 | 0 |
| *Euarchontoglires* | 0 | 0 | 0 | 0 | 0 | 0 | 0 |
| *Eukaryota* | 0 | 0 | 0 | 0 | 0 | 0 | 0 |
| *Eumetazoa* | 0 | 0 | 0 | 0 | 0 | 0 | 0 |
| *Eurotiales* | 0 | 0 | 0 | 0 | 0 | 0 | 0 |
| *Euteleostomi* | 0 | 0 | 0 | 0 | 0 | 0 | 0 |
| *Fagales* | 0 | 0 | 0 | 0 | 0 | 0 | 0 |
| *Fungi* | 0 | 0 | 0 | 0 | 0 | 0 | 0 |
| *Fusarium_graminearum* | 0 | 0 | 0 | 0 | 0 | 0 | 0 |
| *Fusarium_sambucinum_species_complex* | 0 | 0 | 0 | 0 | 0 | 0 | 0 |
| *Gallus_gallus* | 0 | 0 | 0 | 0 | 0 | 0 | 0 |
| *Geospiza_fortis* | 0 | 0 | 0 | 0 | 0 | 0 | 0 |
| *Heterocephalus_glaber* | 0 | 0 | 0 | 0 | 0 | 0 | 0 |
| *Hominidae* | 0 | 0 | 0 | 0 | 0 | 0 | 0 |
| *Homininae* | 0 | 0 | 0 | 0 | 0 | 0 | 5 |
| *Hominoidea* | 0 | 0 | 0 | 0 | 0 | 0 | 1 |
| *Homo_sapiens* | 0 | 0 | 0 | 0 | 0 | 0 | 20 |
| *Hypocreales* | 0 | 0 | 0 | 0 | 0 | 0 | 0 |
| *Ictidomys_tridecemlineatus* | 0 | 0 | 0 | 0 | 0 | 0 | 0 |
| *Kockovaella* | 0 | 0 | 0 | 0 | 0 | 0 | 0 |
| *Lasthenia_californica* | 0 | 0 | 0 | 0 | 0 | 0 | 0 |
| *Lynx_canadensis* | 0 | 0 | 0 | 0 | 0 | 0 | 0 |
| *Macaca_fascicularis* | 0 | 0 | 0 | 0 | 0 | 0 | 0 |
| *Malassezia_restricta* | 0 | 0 | 0 | 0 | 0 | 0 | 0 |
| *Malus_domestica* | 0 | 0 | 0 | 0 | 0 | 0 | 0 |
| *Metarhizium_robertsii* | 0 | 0 | 0 | 0 | 0 | 0 | 0 |
| *Micractinium_conductrix* | 0 | 0 | 0 | 0 | 0 | 0 | 0 |
| *Microascales* | 0 | 0 | 0 | 0 | 0 | 0 | 0 |
| *Mucorales* | 0 | 0 | 0 | 0 | 0 | 0 | 0 |
| *Neognathae* | 0 | 0 | 0 | 0 | 0 | 0 | 0 |
| *Nippostrongylus_brasiliensis* | 0 | 0 | 0 | 0 | 0 | 0 | 0 |
| *Odocoileus* | 0 | 1 | 0 | 0 | 0 | 0 | 0 |
| *Onchocerca_ochengi* | 0 | 0 | 0 | 0 | 0 | 0 | 0 |
| *Oncorhynchus_tshawytscha* | 0 | 0 | 0 | 0 | 0 | 0 | 0 |
| *Opegrapha_vulgata* | 0 | 0 | 0 | 0 | 0 | 0 | 0 |
| *Opisthokonta* | 0 | 0 | 0 | 0 | 0 | 0 | 0 |
| *Oreocharis_mileensis* | 0 | 0 | 0 | 0 | 0 | 0 | 0 |
| *Oryza_sativa* | 0 | 0 | 0 | 0 | 0 | 0 | 0 |
| *Ovis_canadensis* | 0 | 0 | 0 | 0 | 0 | 0 | 0 |
| *Pan_troglodytes* | 0 | 0 | 0 | 0 | 0 | 0 | 0 |
| *Passeriformes* | 0 | 0 | 0 | 0 | 0 | 0 | 0 |
| *Penicillium* | 0 | 0 | 0 | 0 | 0 | 0 | 0 |
| *Pentapetalae* | 0 | 0 | 0 | 1 | 0 | 0 | 0 |
| *Pezizomycotina* | 0 | 0 | 0 | 0 | 0 | 0 | 0 |
| *Phyllostomidae* | 0 | 0 | 0 | 0 | 0 | 0 | 0 |
| *Physcomitrella_patens* | 0 | 0 | 0 | 0 | 0 | 0 | 0 |
| *Pinus* | 0 | 0 | 0 | 0 | 0 | 0 | 0 |
| *Pinus* | 0 | 0 | 0 | 0 | 0 | 0 | 0 |
| *Platyhelminthes* | 0 | 0 | 0 | 0 | 0 | 0 | 0 |
| *Pleosporales* | 0 | 0 | 0 | 0 | 0 | 0 | 0 |
| *Poales* | 0 | 0 | 0 | 0 | 0 | 0 | 0 |
| *Pongo_abelii* | 0 | 0 | 0 | 0 | 0 | 0 | 0 |
| *Populus_trichocarpa* | 0 | 0 | 0 | 0 | 0 | 0 | 0 |
| *Proasellus_solanasi* | 0 | 0 | 0 | 0 | 0 | 0 | 0 |
| *Pterygota* | 0 | 0 | 0 | 0 | 0 | 0 | 0 |
| *Saccharomyces_cerevisiae* | 0 | 0 | 0 | 0 | 0 | 0 | 0 |
| *Saccharomycetales* | 0 | 0 | 0 | 0 | 0 | 0 | 0 |
| *Salarias_fasciatus* | 0 | 0 | 0 | 0 | 0 | 0 | 0 |
| *Scaptodrosophila_lebanonensis* | 0 | 0 | 0 | 0 | 0 | 0 | 0 |
| *Sciaroidea* | 0 | 0 | 0 | 0 | 0 | 0 | 0 |
| *Simiiformes* | 0 | 0 | 0 | 0 | 0 | 0 | 0 |
| *Sordariomycetes* | 0 | 0 | 0 | 0 | 0 | 0 | 0 |
| *Spermatophyta* | 0 | 0 | 0 | 0 | 0 | 0 | 0 |
| *Sporidiobolaceae* | 0 | 0 | 0 | 0 | 0 | 0 | 0 |
| *Sporisorium_graminicola* | 0 | 0 | 0 | 0 | 0 | 0 | 0 |
| *Stereum_hirsutum* | 0 | 0 | 0 | 0 | 0 | 0 | 0 |
| *Stramenopiles* | 0 | 0 | 0 | 0 | 0 | 0 | 0 |
| *Streptopelia_turtur* | 0 | 0 | 0 | 1 | 0 | 0 | 0 |
| *Sturnus* | 0 | 0 | 0 | 0 | 0 | 0 | 0 |
| *Sturnus_vulgaris* | 0 | 0 | 0 | 1 | 0 | 0 | 0 |
| *Tetrapoda* | 0 | 0 | 0 | 0 | 0 | 0 | 0 |
| *Theropithecus_gelada* | 0 | 0 | 0 | 0 | 0 | 0 | 0 |
| *Trebouxiophyceae* | 0 | 0 | 0 | 0 | 0 | 0 | 0 |
| *Tremellales* | 0 | 0 | 0 | 0 | 0 | 0 | 0 |
| *Tremellomycetes* | 0 | 0 | 0 | 0 | 0 | 0 | 0 |
| *Triticinae* | 0 | 0 | 0 | 0 | 0 | 0 | 0 |
| *Triticum* | 0 | 0 | 0 | 0 | 0 | 0 | 0 |
| *Triticum_aestivum* | 0 | 0 | 0 | 0 | 0 | 0 | 0 |
| *Vulpes_vulpes* | 0 | 0 | 0 | 0 | 0 | 0 | 0 |
| *Zea_mays* | 0 | 0 | 0 | 0 | 0 | 0 | 0 |
| *leotiomyceta* | 0 | 0 | 0 | 0 | 0 | 0 | 0 |
| **Planktonbaits1 - Default** | **HYB18SV9_A21980_EBC** | **HYB18SV9_A22301_EBC** | **HYB18SV9_A22302_EBC** | **HYB18SV9_A23351_EBC** | **HYB18SV9_A23352_EBC** | **HYB18SV9_A24029_EBC** | **HYB18SV9_A24030_EBC** |
| *Acrogymnospermae* | 0 | 0 | 0 | 1 | 0 | 0 | 0 |
| *Agaricomycetes_incertae_sedis* | 0 | 0 | 0 | 3 | 0 | 0 | 0 |
| *Agaricomycotina* | 0 | 0 | 0 | 0 | 0 | 0 | 6 |
| *Alternaria_solani* | 0 | 0 | 0 | 1 | 0 | 0 | 0 |
| *Amniota* | 0 | 0 | 0 | 1 | 0 | 0 | 3 |
| *Amygdaloideae* | 0 | 0 | 0 | 0 | 0 | 1 | 0 |
| *Aquila_chrysaetos* | 0 | 0 | 0 | 8 | 0 | 0 | 0 |
| *Ascomycota* | 0 | 0 | 0 | 1 | 0 | 0 | 0 |
| *Aureobasidium_pullulans* | 0 | 0 | 0 | 2 | 0 | 0 | 0 |
| *Aves* | 0 | 0 | 0 | 1 | 0 | 0 | 0 |
| *Basidiomycota* | 0 | 0 | 0 | 1 | 0 | 0 | 5 |
| *Bilateria* | 0 | 1 | 0 | 1 | 1 | 3 | 110 |
| *Boreoeutheria* | 0 | 0 | 0 | 0 | 0 | 1 | 6 |
| *Bos* | 0 | 0 | 0 | 0 | 0 | 0 | 0 |
| *Bos_indicus* | 0 | 0 | 0 | 0 | 0 | 0 | 0 |
| *Bos_indicus_x_Bos_taurus* | 0 | 0 | 0 | 0 | 0 | 0 | 0 |
| *Bos_mutus* | 0 | 0 | 0 | 0 | 0 | 0 | 0 |
| *Bos_taurus* | 0 | 0 | 0 | 0 | 0 | 0 | 0 |
| *Bovidae* | 0 | 0 | 0 | 0 | 0 | 0 | 0 |
| *Bovinae* | 0 | 0 | 0 | 0 | 0 | 0 | 0 |
| *Brugia_timori* | 0 | 0 | 0 | 1 | 0 | 0 | 0 |
| *Camelus_ferus* | 0 | 0 | 0 | 0 | 0 | 2 | 0 |
| *Camponotus_floridanus* | 0 | 2 | 0 | 0 | 0 | 0 | 0 |
| *Catarrhini* | 0 | 0 | 1 | 0 | 0 | 0 | 43 |
| *Cercomonadida* | 0 | 0 | 0 | 1 | 0 | 0 | 0 |
| *Cercopithecinae* | 0 | 0 | 0 | 0 | 0 | 0 | 3 |
| *Cercozoa* | 0 | 0 | 0 | 2 | 0 | 0 | 0 |
| *Chlamydomonadales* | 0 | 0 | 0 | 22 | 0 | 0 | 0 |
| *Coccomyxa* | 0 | 0 | 0 | 1 | 0 | 0 | 0 |
| *Coccomyxa_subellipsoidea* | 0 | 0 | 0 | 1 | 0 | 0 | 0 |
| *Columba* | 0 | 0 | 0 | 1 | 0 | 0 | 0 |
| *Columba_livia* | 0 | 0 | 0 | 3 | 0 | 0 | 0 |
| *Columbidae* | 0 | 0 | 0 | 2 | 0 | 0 | 0 |
| *Cryptococcus_neoformans* | 0 | 0 | 0 | 0 | 0 | 0 | 44 |
| *Culex_pipiens* | 0 | 0 | 0 | 2 | 0 | 0 | 0 |
| *Cyanistes_caeruleus* | 0 | 0 | 0 | 1 | 0 | 0 | 0 |
| *Cyprinus_carpio* | 0 | 0 | 4 | 0 | 8 | 51 | 4 |
| *Daucus_carota* | 0 | 0 | 0 | 3 | 0 | 0 | 0 |
| *Deuterostomia* | 0 | 0 | 0 | 1 | 0 | 0 | 0 |
| *Diaspididae* | 0 | 0 | 0 | 1 | 0 | 0 | 0 |
| *Dioon* | 0 | 0 | 0 | 0 | 0 | 81 | 0 |
| *Dioscorea_rotundata* | 0 | 0 | 0 | 1 | 0 | 0 | 0 |
| *Diphyllobothrium* | 0 | 3 | 0 | 0 | 0 | 73 | 0 |
| *Dothideomycetes* | 0 | 0 | 0 | 2 | 0 | 0 | 0 |
| *Drosophila_pseudoobscura* | 0 | 0 | 0 | 0 | 0 | 1 | 0 |
| *Embryophyta* | 0 | 0 | 0 | 0 | 0 | 7 | 0 |
| *Euarchontoglires* | 0 | 0 | 0 | 0 | 0 | 1 | 0 |
| *Eukaryota* | 0 | 0 | 0 | 2 | 0 | 18 | 8 |
| *Eumetazoa* | 0 | 0 | 2 | 0 | 0 | 0 | 0 |
| *Eurotiales* | 0 | 0 | 0 | 1 | 0 | 0 | 0 |
| *Euteleostomi* | 0 | 0 | 0 | 0 | 0 | 4 | 0 |
| *Fagales* | 0 | 0 | 0 | 14 | 0 | 0 | 0 |
| *Fungi* | 0 | 0 | 0 | 2 | 0 | 0 | 11 |
| *Fusarium_graminearum* | 0 | 0 | 0 | 1 | 0 | 0 | 0 |
| *Fusarium_sambucinum_species_complex* | 0 | 0 | 0 | 1 | 0 | 0 | 0 |
| *Gallus_gallus* | 0 | 0 | 1 | 0 | 0 | 0 | 0 |
| *Geospiza_fortis* | 0 | 0 | 0 | 1 | 0 | 0 | 0 |
| *Heterocephalus_glaber* | 0 | 0 | 1 | 0 | 0 | 0 | 0 |
| *Hominidae* | 0 | 0 | 0 | 0 | 0 | 0 | 45 |
| *Homininae* | 1 | 3 | 1 | 1 | 0 | 0 | 144 |
| *Hominoidea* | 0 | 0 | 0 | 0 | 0 | 0 | 12 |
| *Homo_sapiens* | 1 | 3 | 0 | 5 | 3 | 1 | 650 |
| *Hypocreales* | 0 | 0 | 0 | 0 | 0 | 0 | 1 |
| *Ictidomys_tridecemlineatus* | 1 | 3 | 5 | 0 | 1 | 15 | 0 |
| *Kockovaella* | 0 | 0 | 0 | 3 | 0 | 0 | 0 |
| *Lasthenia_californica* | 0 | 0 | 0 | 0 | 0 | 3 | 0 |
| *Lynx_canadensis* | 0 | 0 | 0 | 0 | 0 | 0 | 1 |
| *Macaca_fascicularis* | 0 | 0 | 0 | 0 | 0 | 0 | 1 |
| *Malassezia_restricta* | 0 | 0 | 0 | 0 | 0 | 0 | 2 |
| *Malus_domestica* | 0 | 0 | 0 | 0 | 0 | 0 | 2 |
| *Metarhizium_robertsii* | 0 | 0 | 0 | 0 | 0 | 0 | 1 |
| *Micractinium_conductrix* | 0 | 0 | 0 | 1 | 0 | 0 | 0 |
| *Microascales* | 0 | 0 | 0 | 1 | 0 | 0 | 0 |
| *Mucorales* | 0 | 0 | 0 | 1 | 0 | 0 | 0 |
| *Neognathae* | 0 | 0 | 0 | 6 | 0 | 0 | 0 |
| *Nippostrongylus_brasiliensis* | 0 | 0 | 0 | 0 | 1 | 0 | 0 |
| *Odocoileus* | 0 | 2 | 0 | 0 | 0 | 0 | 0 |
| *Onchocerca_ochengi* | 0 | 0 | 0 | 1 | 0 | 0 | 0 |
| *Oncorhynchus_tshawytscha* | 0 | 0 | 0 | 3 | 0 | 0 | 0 |
| *Opegrapha_vulgata* | 0 | 0 | 0 | 0 | 0 | 0 | 1 |
| *Opisthokonta* | 0 | 0 | 0 | 0 | 0 | 0 | 2 |
| *Oreocharis_mileensis* | 0 | 0 | 0 | 1 | 0 | 0 | 0 |
| *Oryza_sativa* | 0 | 0 | 0 | 1 | 0 | 0 | 0 |
| *Ovis_canadensis* | 0 | 1 | 0 | 0 | 0 | 0 | 0 |
| *Pan_troglodytes* | 0 | 0 | 0 | 0 | 0 | 0 | 17 |
| *Passeriformes* | 0 | 0 | 0 | 8 | 0 | 0 | 0 |
| *Penicillium* | 0 | 0 | 0 | 2 | 0 | 0 | 0 |
| *Pentapetalae* | 0 | 0 | 0 | 2 | 0 | 0 | 0 |
| *Pezizomycotina* | 0 | 0 | 0 | 2 | 0 | 0 | 0 |
| *Phyllostomidae* | 0 | 0 | 0 | 1 | 0 | 0 | 0 |
| *Physcomitrella_patens* | 0 | 0 | 0 | 0 | 0 | 1 | 0 |
| *Pinus* | 0 | 0 | 0 | 0 | 0 | 0 | 0 |
| *Pinus* | 0 | 0 | 0 | 1 | 0 | 0 | 0 |
| *Platyhelminthes* | 0 | 0 | 0 | 0 | 0 | 0 | 1 |
| *Pleosporales* | 0 | 0 | 0 | 2 | 0 | 0 | 0 |
| *Poales* | 0 | 1 | 0 | 0 | 0 | 0 | 0 |
| *Pongo_abelii* | 0 | 0 | 0 | 0 | 0 | 4 | 1 |
| *Populus_trichocarpa* | 0 | 0 | 0 | 2 | 0 | 0 | 0 |
| *Proasellus_solanasi* | 0 | 0 | 0 | 0 | 0 | 1 | 0 |
| *Pterygota* | 0 | 0 | 0 | 1 | 0 | 0 | 0 |
| *Saccharomyces_cerevisiae* | 0 | 1 | 0 | 0 | 0 | 0 | 0 |
| *Saccharomycetales* | 0 | 0 | 0 | 0 | 0 | 0 | 2 |
| *Salarias_fasciatus* | 0 | 0 | 1 | 0 | 0 | 1 | 0 |
| *Scaptodrosophila_lebanonensis* | 0 | 0 | 0 | 0 | 0 | 1 | 0 |
| *Sciaroidea* | 0 | 0 | 0 | 1 | 0 | 0 | 0 |
| *Simiiformes* | 0 | 0 | 0 | 0 | 0 | 0 | 8 |
| *Sordariomycetes* | 0 | 0 | 0 | 1 | 0 | 0 | 0 |
| *Spermatophyta* | 0 | 0 | 0 | 0 | 0 | 2 | 0 |
| *Sporidiobolaceae* | 0 | 0 | 0 | 0 | 0 | 0 | 1 |
| *Sporisorium_graminicola* | 0 | 0 | 0 | 1 | 0 | 0 | 0 |
| *Stereum_hirsutum* | 0 | 0 | 0 | 5 | 0 | 0 | 0 |
| *Stramenopiles* | 0 | 0 | 0 | 6 | 0 | 0 | 0 |
| *Streptopelia_turtur* | 0 | 0 | 0 | 29 | 0 | 0 | 0 |
| *Sturnus* | 0 | 0 | 0 | 1 | 0 | 0 | 0 |
| *Sturnus_vulgaris* | 0 | 0 | 0 | 5 | 0 | 0 | 0 |
| *Tetrapoda* | 0 | 0 | 0 | 1 | 0 | 0 | 1 |
| *Theropithecus_gelada* | 0 | 0 | 0 | 0 | 0 | 0 | 1 |
| *Trebouxiophyceae* | 0 | 0 | 0 | 1 | 0 | 0 | 0 |
| *Tremellales* | 0 | 0 | 0 | 0 | 0 | 0 | 1 |
| *Tremellomycetes* | 0 | 0 | 0 | 2 | 0 | 0 | 0 |
| *Triticinae* | 0 | 1 | 0 | 0 | 0 | 0 | 0 |
| *Triticum* | 0 | 1 | 0 | 0 | 0 | 0 | 1 |
| *Triticum_aestivum* | 0 | 0 | 0 | 0 | 0 | 0 | 1 |
| *Vulpes_vulpes* | 0 | 0 | 0 | 0 | 0 | 0 | 1 |
| *Zea_mays* | 0 | 0 | 0 | 2 | 0 | 0 | 0 |
| *leotiomyceta* | 0 | 0 | 0 | 3 | 0 | 0 | 3 |
| **HABbaits1 - Ancient** | **HYBHAB_A21980_EBC** | **HYBHAB_A22301_EBC** | **HYBHAB_A22302_EBC** | **HYBHAB_A23351_EBC** | **HYBHAB_A23352_EBC** | **HYBHAB_A24029_EBC** | **HYBHAB_A24030_EBC** |
| *Agaricomycetes* | 0 | 0 | 0 | 0 | 0 | 0 | 0 |
| *Agaricomycetes_incertae_sedis* | 0 | 0 | 0 | 0 | 0 | 0 | 0 |
| *Amniota* | 0 | 0 | 0 | 0 | 0 | 0 | 0 |
| *Aquila_chrysaetos* | 0 | 0 | 0 | 0 | 0 | 0 | 0 |
| *Ascomycota* | 0 | 0 | 0 | 0 | 0 | 0 | 0 |
| *Basidiomycota* | 0 | 0 | 0 | 0 | 0 | 0 | 0 |
| *Bilateria* | 0 | 0 | 0 | 0 | 0 | 0 | 1 |
| *Boreoeutheria* | 0 | 0 | 0 | 0 | 0 | 0 | 0 |
| *Bos* | 0 | 0 | 0 | 0 | 0 | 0 | 0 |
| *Bos_indicus* | 0 | 0 | 0 | 0 | 0 | 0 | 0 |
| *Bos_indicus_x_Bos_taurus* | 0 | 0 | 0 | 0 | 0 | 0 | 0 |
| *Bos_mutus* | 0 | 0 | 0 | 0 | 0 | 0 | 0 |
| *Bos_taurus* | 0 | 0 | 0 | 0 | 0 | 0 | 0 |
| *Bovidae* | 0 | 0 | 0 | 0 | 0 | 0 | 0 |
| *Bovinae* | 0 | 0 | 0 | 0 | 0 | 0 | 0 |
| *Catarrhini* | 0 | 0 | 0 | 0 | 0 | 0 | 0 |
| *Cercozoa* | 0 | 0 | 0 | 0 | 0 | 0 | 0 |
| *Chlorophyta* | 0 | 0 | 0 | 0 | 0 | 0 | 0 |
| *Corvus_brachyrhynchos* | 0 | 0 | 0 | 0 | 0 | 0 | 0 |
| *Cyprinus_carpio* | 0 | 0 | 0 | 0 | 0 | 0 | 1 |
| *Diaspididae* | 0 | 0 | 0 | 0 | 0 | 0 | 0 |
| *Dioon* | 0 | 0 | 0 | 0 | 0 | 0 | 0 |
| *Diphyllobothrium* | 0 | 0 | 0 | 0 | 0 | 0 | 0 |
| *Dothideomycetes* | 0 | 0 | 0 | 0 | 0 | 0 | 0 |
| *Embryophyta* | 0 | 0 | 0 | 0 | 0 | 0 | 0 |
| *Euarchontoglires* | 0 | 0 | 0 | 0 | 0 | 0 | 0 |
| *Eukaryota* | 0 | 0 | 0 | 0 | 0 | 0 | 0 |
| *Eurotiales* | 0 | 0 | 0 | 0 | 0 | 0 | 0 |
| *Euteleostomi* | 0 | 0 | 0 | 0 | 0 | 0 | 0 |
| *Fungi* | 0 | 0 | 0 | 0 | 0 | 0 | 0 |
| *Harmonia_axyridis* | 0 | 0 | 0 | 0 | 0 | 0 | 0 |
| *Hominidae* | 0 | 0 | 0 | 0 | 0 | 0 | 0 |
| *Homininae* | 0 | 0 | 0 | 0 | 0 | 0 | 0 |
| *Hominoidea* | 0 | 0 | 0 | 0 | 0 | 0 | 0 |
| *Homo_sapiens* | 0 | 0 | 0 | 0 | 0 | 0 | 1 |
| *Lynx_canadensis* | 0 | 0 | 0 | 0 | 0 | 0 | 0 |
| *Malassezia_restricta* | 0 | 0 | 0 | 0 | 0 | 0 | 0 |
| *Malus_domestica* | 0 | 0 | 0 | 0 | 0 | 0 | 0 |
| *Mesangiospermae* | 0 | 0 | 0 | 0 | 0 | 0 | 0 |
| *Neognathae* | 0 | 0 | 0 | 0 | 0 | 0 | 0 |
| *Opegrapha_vulgata* | 0 | 0 | 0 | 0 | 0 | 0 | 0 |
| *Paraneoptera* | 0 | 0 | 0 | 0 | 0 | 0 | 0 |
| *Passeriformes* | 0 | 0 | 0 | 0 | 0 | 0 | 0 |
| *Pentapetalae* | 0 | 0 | 0 | 0 | 0 | 0 | 0 |
| *Pleosporales* | 0 | 0 | 0 | 0 | 0 | 0 | 0 |
| *Pongo_abelii* | 0 | 0 | 0 | 0 | 0 | 0 | 0 |
| *Rhabditidae* | 0 | 0 | 0 | 0 | 0 | 0 | 0 |
| *Rhizophydiales* | 0 | 0 | 0 | 0 | 0 | 0 | 0 |
| *Sciaroidea* | 0 | 0 | 0 | 0 | 0 | 0 | 0 |
| *Stenamoeba* | 0 | 0 | 0 | 0 | 0 | 0 | 0 |
| *Stramenopiles* | 0 | 0 | 0 | 0 | 0 | 0 | 0 |
| *Streptopelia_turtur* | 0 | 0 | 0 | 0 | 0 | 0 | 0 |
| *Theropithecus_gelada* | 0 | 0 | 0 | 0 | 0 | 0 | 0 |
| *Triticum* | 0 | 0 | 0 | 0 | 0 | 0 | 0 |
| *Triticum_aestivum* | 0 | 0 | 0 | 0 | 0 | 0 | 0 |
| *Zonotrichia_albicollis* | 0 | 0 | 0 | 0 | 0 | 0 | 0 |
| *leotiomyceta* | 0 | 0 | 0 | 0 | 0 | 0 | 0 |
| **HABbaits1 - Default** | **HYBHAB_A21980_EBC** | **HYBHAB_A22301_EBC** | **HYBHAB_A22302_EBC** | **HYBHAB_A23351_EBC** | **HYBHAB_A23352_EBC** | **HYBHAB_A24029_EBC** | **HYBHAB_A24030_EBC** |
| *Agaricomycetes* | 0 | 0 | 0 | 1 | 0 | 0 | 0 |
| *Agaricomycetes_incertae_sedis* | 0 | 0 | 0 | 1 | 0 | 0 | 0 |
| *Amniota* | 0 | 0 | 0 | 1 | 0 | 0 | 0 |
| *Aquila_chrysaetos* | 0 | 0 | 0 | 1 | 0 | 0 | 0 |
| *Ascomycota* | 0 | 0 | 0 | 2 | 0 | 0 | 0 |
| *Basidiomycota* | 0 | 0 | 0 | 0 | 0 | 0 | 1 |
| *Bilateria* | 0 | 0 | 0 | 0 | 0 | 1 | 13 |
| *Boreoeutheria* | 0 | 0 | 0 | 0 | 0 | 0 | 1 |
| *Bos* | 0 | 0 | 0 | 0 | 0 | 0 | 0 |
| *Bos_indicus* | 0 | 0 | 0 | 0 | 0 | 0 | 0 |
| *Bos_indicus_x_Bos_taurus* | 0 | 0 | 0 | 0 | 0 | 0 | 0 |
| *Bos_mutus* | 0 | 0 | 0 | 0 | 0 | 0 | 0 |
| *Bos_taurus* | 0 | 0 | 0 | 0 | 0 | 0 | 0 |
| *Bovidae* | 0 | 0 | 0 | 0 | 0 | 0 | 0 |
| *Bovinae* | 0 | 0 | 0 | 0 | 0 | 0 | 0 |
| *Catarrhini* | 0 | 0 | 0 | 0 | 0 | 0 | 5 |
| *Cercozoa* | 0 | 0 | 0 | 2 | 0 | 0 | 0 |
| *Chlorophyta* | 0 | 0 | 0 | 1 | 0 | 0 | 0 |
| *Corvus_brachyrhynchos* | 0 | 0 | 0 | 1 | 0 | 0 | 0 |
| *Cyprinus_carpio* | 0 | 0 | 0 | 0 | 3 | 2 | 1 |
| *Diaspididae* | 0 | 0 | 0 | 1 | 0 | 0 | 0 |
| *Dioon* | 0 | 0 | 0 | 0 | 0 | 5 | 0 |
| *Diphyllobothrium* | 0 | 1 | 0 | 0 | 0 | 3 | 0 |
| *Dothideomycetes* | 0 | 0 | 0 | 3 | 0 | 0 | 0 |
| *Embryophyta* | 0 | 0 | 0 | 0 | 0 | 1 | 0 |
| *Euarchontoglires* | 0 | 0 | 0 | 0 | 0 | 1 | 0 |
| *Eukaryota* | 0 | 0 | 0 | 2 | 0 | 0 | 1 |
| *Eurotiales* | 0 | 0 | 0 | 1 | 0 | 0 | 0 |
| *Euteleostomi* | 0 | 0 | 0 | 0 | 0 | 1 | 0 |
| *Fungi* | 0 | 0 | 0 | 1 | 0 | 0 | 0 |
| *Harmonia_axyridis* | 0 | 0 | 0 | 1 | 0 | 0 | 0 |
| *Hominidae* | 0 | 0 | 0 | 0 | 0 | 0 | 7 |
| *Homininae* | 0 | 1 | 0 | 0 | 0 | 0 | 20 |
| *Hominoidea* | 0 | 0 | 0 | 0 | 0 | 0 | 2 |
| *Homo_sapiens* | 0 | 0 | 0 | 1 | 0 | 0 | 83 |
| *Lynx_canadensis* | 0 | 0 | 0 | 0 | 0 | 0 | 1 |
| *Malassezia_restricta* | 0 | 0 | 0 | 0 | 0 | 0 | 1 |
| *Malus_domestica* | 0 | 0 | 0 | 0 | 0 | 0 | 2 |
| *Mesangiospermae* | 0 | 0 | 0 | 2 | 0 | 0 | 0 |
| *Neognathae* | 0 | 0 | 0 | 1 | 0 | 0 | 0 |
| *Opegrapha_vulgata* | 0 | 0 | 0 | 0 | 0 | 0 | 1 |
| *Paraneoptera* | 0 | 0 | 0 | 1 | 0 | 0 | 0 |
| *Passeriformes* | 0 | 0 | 0 | 2 | 0 | 0 | 0 |
| *Pentapetalae* | 0 | 0 | 0 | 3 | 0 | 0 | 0 |
| *Pleosporales* | 0 | 0 | 0 | 1 | 0 | 0 | 0 |
| *Pongo_abelii* | 0 | 0 | 0 | 0 | 0 | 1 | 0 |
| *Rhabditidae* | 0 | 0 | 0 | 1 | 0 | 0 | 0 |
| *Rhizophydiales* | 0 | 0 | 0 | 1 | 0 | 0 | 0 |
| *Sciaroidea* | 0 | 0 | 0 | 1 | 0 | 0 | 0 |
| *Stenamoeba* | 0 | 0 | 0 | 1 | 0 | 0 | 0 |
| *Stramenopiles* | 0 | 0 | 0 | 1 | 0 | 0 | 0 |
| *Streptopelia_turtur* | 0 | 0 | 0 | 1 | 0 | 0 | 0 |
| *Theropithecus_gelada* | 0 | 0 | 0 | 0 | 0 | 0 | 1 |
| *Triticum* | 0 | 0 | 0 | 0 | 0 | 0 | 1 |
| *Triticum_aestivum* | 0 | 0 | 0 | 0 | 0 | 0 | 1 |
| *Zonotrichia_albicollis* | 0 | 0 | 0 | 1 | 0 | 0 | 0 |
| *leotiomyceta* | 0 | 0 | 0 | 1 | 0 | 0 | 0 |

**Supplementary Material Table 3.** Summary of Eukaryota reads classified as ancient and default by HOPS . Listed are the total number of reads of Shotgun, Planktonbaits1 and HABbaits1, as well as proportions. The proportion of ancient reads per sample (in italics) provides a measure of *sed*aDNA damage per sample for eukaryotes (main text Fig. 4). The majority of reads are classified as default in all three datasets Shotgun, Planktonbaits1, and HABbaits1 (total of 3,237, 1,661, and 200 default reads, respectively; compared to a total of 170, 45, and 3 ancient reads), suggesting that these are primarily modern contaminants.

| **Sample ID** | **21931_MCS3_34** | **21798_GC2B_5** | **21802_GC2B_15** | **21804_GC2B_20** | **21806_GC2B_25** | **21808_GC2B_30** | **21810_GC2B_35** | **21816_GC2B_50** | **21822_GC2B_65** |
| --- | --- | --- | --- | --- | --- | --- | --- | --- | --- |
| **Depth (cmbsf)** | **0** | **5** | **15** | **20** | **25** | **30** | **35** | **50** | **65** |
| **Totals (number of reads)** |  |  |  |  |  |  |  |  |  |
| **Shotgun** |  |  |  |  |  |  |  |  |  |
| **Ancient** | 28 | 63 | 49 | 176 | 135 | 789 | 494 | 1,259 | 1,480 |
| **Default** | 615 | 1,067 | 808 | 1,532 | 1,614 | 3,641 | 2,589 | 5,025 | 5,577 |
| **Planktonbaits1** |  |  |  |  |  |  |  |  |  |
| **Ancient** | 179 | 543 | 566 | 2,592 | 365 | 1,161 | 1,504 | 3,529 | 6,546 |
| **Default** | 2,300 | 5,485 | 5,090 | 16,446 | 3,217 | 5,487 | 6,870 | 15,071 | 24,557 |
| **HABbaits1** |  |  |  |  |  |  |  |  |  |
| **Ancient** | 600 | 1,330 | 1,386 | 7,302 | 1,103 | 8,354 | 5,322 | 7,856 | 10,195 |
| **Default** | 7,618 | 14,768 | 12,922 | 45,657 | 9,100 | 36,085 | 25,297 | 33,671 | 37,861 |
| **Proportions (%)** |  |  |  |  |  |  |  |  |  |
| **Shotgun** |  |  |  |  |  |  |  |  |  |
| ***Ancient*** | *4* | *6* | *6* | *10* | *8* | *18* | *16* | *20* | *21* |
| **Default** | 96 | 94 | 94 | 90 | 92 | 82 | 84 | 80 | 79 |
| **Planktonbaits1** |  |  |  |  |  |  |  |  |  |
| ***Ancient*** | *7* | *9* | *10* | *14* | *10* | *17* | *18* | *19* | *21* |
| **Default** | 93 | 91 | 90 | 86 | 90 | 83 | 82 | 81 | 79 |
| **HABbaits1** |  |  |  |  |  |  |  |  |  |
| ***Ancient*** | *7* | *8* | *10* | *14* | *11* | *19* | *17* | *19* | *21* |
| **Default** | 93 | 92 | 90 | 86 | 89 | 81 | 83 | 81 | 79 |
| **Eukaryota Ancient:Default Ratio** |  |  |  |  |  |  |  |  |  |
| **Shotgun** | 0.05 | 0.06 | 0.06 | 0.11 | 0.08 | 0.22 | 0.19 | 0.25 | 0.27 |
| **Planktonbaits1** | 0.08 | 0.10 | 0.11 | 0.16 | 0.11 | 0.21 | 0.22 | 0.23 | 0.27 |
| **HABbaits1** | 0.08 | 0.09 | 0.11 | 0.16 | 0.12 | 0.23 | 0.21 | 0.23 | 0.27 |
|  |  |  |  |  |  |  |  |  |  |
| **Sample ID** | **21828_GC2B_75** | **21832_GC2B_85** | **21838_GC2B_100** | **21856_GC2A_15** | **21868_GC2A_45** | **21876_GC2A_65** | **21888_GC2A_95** | **21896_GC2A_115** | **A21908_GC2A_143** |
| **Depth (cmbsf)** | **75** | **85** | **100** | **139** | **169** | **189** | **219** | **239** | **267** |
| **Totals (number of reads)** |  |  |  |  |  |  |  |  |  |
| **Shotgun** |  |  |  |  |  |  |  |  |  |
| **Ancient** | 1,043 | 3,404 | 1,626 | 1,482 | 829 | 773 | 2,429 | 1,720 | 1,203 |
| **Default** | 4,463 | 11,976 | 6,611 | 4,831 | 3,268 | 2,852 | 7,490 | 5,819 | 4,662 |
| **Planktonbaits1** |  |  |  |  |  |  |  |  |  |
| **Ancient** | 3,467 | 1,085 | 4,319 | 5,530 | 2,402 | 4,266 | 3,173 | 2,630 | 2,507 |
| **Default** | 13,804 | 3,824 | 16,247 | 19,073 | 10,604 | 15,549 | 10,852 | 8,572 | 9,558 |
| **HABbaits1** |  |  |  |  |  |  |  |  |  |
| **Ancient** | 9,407 | 8,706 | 1,994 | 6,787 | 1,096 | 11,863 | 9,566 | 6,259 | 9,708 |
| **Default** | 38,693 | 30,762 | 8,081 | 23,004 | 4,732 | 41,089 | 34,636 | 21,095 | 32,818 |
| **Proportions (%)** |  |  |  |  |  |  |  |  |  |
| **Shotgun** |  |  |  |  |  |  |  |  |  |
| ***Ancient*** | *19* | *22* | *20* | *23* | *20* | *21* | *24* | *23* | *21* |
| **Default** | 81 | 78 | 80 | 77 | 80 | 79 | 76 | 77 | 79 |
| **Planktonbaits1** |  |  |  |  |  |  |  |  |  |
| ***Ancient*** | *20* | *22* | *21* | *22* | *18* | *22* | *23* | *23* | *21* |
| **Default** | 80 | 78 | 79 | 78 | 82 | 78 | 77 | 77 | 79 |
| **HABbaits1** |  |  |  |  |  |  |  |  |  |
| ***Ancient*** | *20* | *22* | *20* | *23* | *19* | *22* | *22* | *23* | *23* |
| **Default** | 80 | 78 | 80 | 77 | 81 | 78 | 78 | 77 | 77 |
| **Eukaryota Ancient:Default Ratio** |  |  |  |  |  |  |  |  |  |
| **Shotgun** | 0.23 | 0.28 | 0.25 | 0.31 | 0.25 | 0.27 | 0.32 | 0.30 | 0.26 |
| **Planktonbaits1** | 0.25 | 0.28 | 0.27 | 0.29 | 0.23 | 0.27 | 0.29 | 0.31 | 0.26 |
| **HABbaits1** | 0.24 | 0.28 | 0.25 | 0.30 | 0.23 | 0.29 | 0.28 | 0.30 | 0.30 |
|  |  |  |  |  |  |  |  |  |  |
| **Sample ID** | **21915_MCS3_2** | **21917_MCS3_4** | **21919_MCS3_6** | **21921_MCS3_12** | **21923_MCS3_15** | **21925_MCS3_20** | **21927_MCS3_25** | **21929_MCS3_30** | **21931_MCS3_34** |
| **Depth (cmbsf)** | **2** | **4** | **6** | **12** | **15** | **20** | **25** | **30** | **34** |
| **Totals (number of reads)** |  |  |  |  |  |  |  |  |  |
| **Shotgun** |  |  |  |  |  |  |  |  |  |
| **Ancient** | 27 | 14 | 20 | 46 | 38 | 32 | 24 | 58 | 62 |
| **Default** | 746 | 620 | 811 | 1,168 | 953 | 853 | 811 | 869 | 935 |
| **Planktonbaits1** |  |  |  |  |  |  |  |  |  |
| **Ancient** | 63 | 3 | 92 | 89 | 92 | 81 | 65 | 223 | 156 |
| **Default** | 1,638 | 409 | 2,916 | 3,160 | 1,897 | 1,527 | 1,459 | 2,386 | 1,652 |
| **HABbaits1** |  |  |  |  |  |  |  |  |  |
| **Ancient** | 257 | 305 | 669 | 520 | 464 | 503 | 461 | 1,093 | 837 |
| **Default** | 6,712 | 8,486 | 16,860 | 14,194 | 8,390 | 8,468 | 7,183 | 11,573 | 7,251 |
| **Proportions (%)** |  |  |  |  |  |  |  |  |  |
| **Shotgun** |  |  |  |  |  |  |  |  |  |
| ***Ancient*** | *3* | *2* | *2* | *4* | *4* | *4* | *3* | *6* | *6* |
| **Default** | 97 | 98 | 98 | 96 | 96 | 96 | 97 | 94 | 94 |
| **Planktonbaits1** |  |  |  |  |  |  |  |  |  |
| ***Ancient*** | *4* | *1* | *3* | *3* | *5* | *5* | *4* | *9* | *9* |
| **Default** | 96 | 99 | 97 | 97 | 95 | 95 | 96 | 91 | 91 |
| **HABbaits1** |  |  |  |  |  |  |  |  |  |
| ***Ancient*** | *4* | *3* | *4* | *4* | *5* | *6* | *6* | *9* | *10* |
| **Default** | 96 | 97 | 96 | 96 | 95 | 94 | 94 | 91 | 90 |
| **Eukaryota Ancient:Default Ratio** |  |  |  |  |  |  |  |  |  |
| **Shotgun** | 0.04 | 0.02 | 0.02 | 0.04 | 0.04 | 0.04 | 0.03 | 0.07 | 0.07 |
| **Planktonbaits1** | 0.04 | 0.01 | 0.03 | 0.03 | 0.05 | 0.05 | 0.04 | 0.09 | 0.09 |
| **HABbaits1** | 0.04 | 0.04 | 0.04 | 0.04 | 0.06 | 0.06 | 0.06 | 0.09 | 0.12 |
